# Complement-Coagulation Cross-talk: Factor H-mediated regulation of the Complement Classical Pathway activation by fibrin clots

**DOI:** 10.3389/fimmu.2024.1368852

**Published:** 2024-06-12

**Authors:** Yu-Hoi Kang, Praveen M. Varghese, Ahmad Al Aiyan, Kirsten Pondman, Uday Kishore, Robert B. Sim

**Affiliations:** ^1^ Medical Research Council Immunochemistry Unit, Department of Biochemistry, University of Oxford, Oxford, United Kingdom; ^2^ MediMabBio Inc., Pangyo Business Growth Centre, Gyeonggi-do, Republic of Korea; ^3^ School of Biosciences and Technology, Vellore Institute of Technology, Vellore, India; ^4^ Department of Veterinary Medicine (CAVM), United Arab Emirates University, Al Ain, United Arab Emirates; ^5^ Applied Microfluidics for BioEngineering Research, MESA+ Institute for Nanotechnology & TechMed Centre, University of Twente, Enschede, Netherlands; ^6^ Zayed Centre for Health Sciences, United Arab Emirates University, Al Ain, United Arab Emirates

**Keywords:** complement, coagulation, classical pathway, C1q, factor H, regulation, COVID-19

## Abstract

The classical pathway of the complement system is activated by the binding of C1q in the C1 complex to the target activator, including immune complexes. Factor H is regarded as the key downregulatory protein of the complement alternative pathway. However, both C1q and factor H bind to target surfaces via charge distribution patterns. For a few targets, C1q and factor H compete for binding to common or overlapping sites. Factor H, therefore, can effectively regulate the classical pathway activation through such targets, in addition to its previously characterized role in the alternative pathway. Both C1q and factor H are known to recognize foreign or altered-self materials, e.g., bacteria, viruses, and apoptotic/necrotic cells. Clots, formed by the coagulation system, are an example of altered self. Factor H is present abundantly in platelets and is a well-known substrate for FXIIIa. Here, we investigated whether clots activate the complement classical pathway and whether this is regulated by factor H. We show here that both C1q and factor H bind to the fibrin formed in microtiter plates and the fibrin clots formed under *in vitro* physiological conditions. Both C1q and factor H become covalently bound to fibrin clots, and this is mediated via FXIIIa. We also show that fibrin clots activate the classical pathway of complement, as demonstrated by C4 consumption and membrane attack complex detection assays. Thus, factor H downregulates the activation of the classical pathway induced by fibrin clots. These results elucidate the intricate molecular mechanisms through which the complement and coagulation pathways intersect and have regulatory consequences.

## Introduction

**Figure 1:**
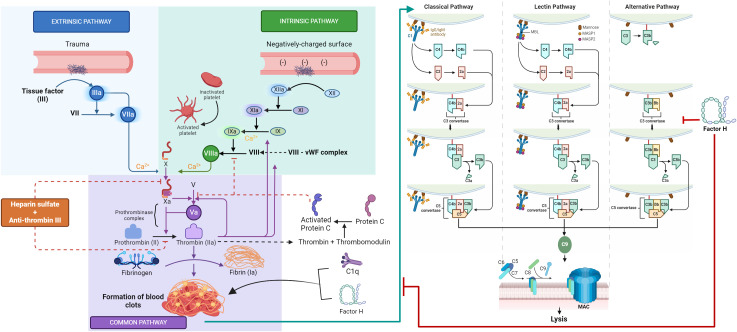
Novel cross-talk between the coagulation and complement systems. This graphic abstract summarizes the distinct yet interconnected mechanisms of the complement and coagulation pathways. The complement classical pathway is initiated by the binding of C1q to IgG- or IgM-containing immune complexes or other non-immunoglobulin targets, while the lectin pathway is activated when mannan-binding lectin (MBL) encounters conserved pathogenic carbohydrate motifs, leading to the activation of the MBL-associated serine proteases (MASPs). Activation of the classical or lectin pathways results in the cleavage of C4 and C2, producing the C3 convertase (C4b2a), which cleaves C3 to form C3b. In the alternative pathway, spontaneous hydrolysis of C3 yields a C3b-like molecule C3(H_2_O), which binds to **f** actor B, facilitating its cleavage by **f** actor D to produce Bb. The resultant C3(H_2_O)Bb, analogous to C3 convertase C4b2a, cleaves C3 to generate C3b. The **c** ovalently bound C3b on target surfaces forms more convertase, C3bBb, and the association of C3b with either C4b2a or C3bBb transforms them into classical or alternative pathway C5 convertases, respectively. The cleavage of C5 by these convertases initiates the assembly of the membrane attack complex (MAC, or C5b-9), which binds to microbial surfaces, potentially leading to lipid bilayer membrane lysis. The coagulation pathway is initiated by vascular wall damage and is subdivided into intrinsic, extrinsic, and common pathways. The extrinsic pathway is triggered by tissue factor exposure, activating factor VIIa and calcium. The intrinsic pathway starts with factor XII exposure to subendothelial collagen, setting off an activation cascade involving factors XI, IX, and VIII. Both pathways culminate in factor X activation, marking the onset of the common pathway. Factor X, alongside factor V, forms the prothrombinase complex, catalyzing the conversion of prothrombin into thrombin, which then mediates the polymerization of fibrinogen into fibrin monomers, cross-linked by factor XIII to form a stable fibrin clot, which is able to capture platelets and red blood cells, effectively sealing the wound and stemming plasma loss. The coagulation cascade and the complement system communicate through many direct and bidirectional interactions. A novel interaction described in this study is between factor H, C1q, and fibrin clots. Factor H and C1q have been shown to covalently bind to fibrin clots with high specificity. FXIIIa aids in cross-linking factor H and C1q into the clots. Moreover, clots activate the complement classical pathway, with factor H dampening this activation, suggesting a regulatory role for factor H in the clot-induced activation of the classical pathway. The figure was created using templates provided at Biorender.com.

The coagulation and the complement pathways are essential systems that help achieve homeostasis. The complement system is a major constituent of the innate immune system, while the coagulation system is a main actor in hemostasis. Both pathways are known to be activated in the case of injuries or in the presence of pathogens. Several studies have established that these two proteolytic pathways are intertwined (1, 2) and thus influence each other’s initiation, effects, and endpoints (**Figure 1**) (3). The innate immune system is characterized by its ability to distinguish between self, non-self, and altered self. The complement system plays a crucial part in the innate immune surveillance and is activated via three pathways: classical, alternative, and lectin (4). The classical pathway is activated through the binding of C1q to immunoglobulin G (IgG)- or IgM-containing immune complexes or other non-immunoglobulin targets. These include nucleic acids and chromatin, mitochondrial membranes, possibly via cardiolipin or mitochondrial proteins, fibronectin, some viruses, Gram-positive bacteria via capsular polysaccharides, and Gram-negative bacteria via the lipid A component of the lipopolysaccharide (5). There is considerable interest in the role of complement in clearing apoptotic cells, which has been suggested to occur through the direct interaction of C1q with altered phospholipid distribution on these cells, including that of phosphatidylserine (6). The binding of C1q to the target induces a conformational change in C1q, which leads to the activation of the serine protease proenzyme C1r, which then activates the proenzyme C1s, initiating the classical pathway (7). The activation of the classical (or the lectin pathway) leads to the cleavage of C4 and C2, yielding C3 convertase (C4b2a), which then cleaves C3 to form C3b. For the alternative pathway, C3 is spontaneously hydrolyzed to a C3b-like form [C3(H_2_O)] due to the hydrolysis of the internal thioester bond. C3(H_2_O) binds to factor B, which enables factor D to cleave factor B to Bb. This then forms C3(H_2_O)Bb, which is homologous to the C3 convertase, C4b2a. C3(H_2_O)Bb then cleaves C3 to C3b, and C3b can bind covalently to target surfaces, on which it forms more convertase, C3bBb (8). The binding of C3b to C4b2a or C3bBb converts them into classical or alternative pathway C5 convertases, respectively. The subsequent cleavage of C5 by the C5 convertase initiates the formation of the membrane attack complex (MAC, or C5b-9). This complex binds to the microbial surface and can cause lysis of the lipid bilayer membranes (4).

Dysregulated activation of the complement system can cause permanent tissue or organ damage. Hence, the complement system is kept in check by various regulatory proteins to limit undesired inflammatory responses and tissue damage. For example, factor H regulates the activation of the alternative pathway by binding soluble or membrane-bound C3b and acting as a decay-accelerating factor for C3bBb (9) or a non-enzymatic cofactor for the cleavage of C3b to iC3b by factor I (10, 11). C1q and factor H compete with each other for the same or overlapping binding sites, such as anionic phospholipids, lipid A, and whole *Escherichia coli* (12–14), suggesting that factor H is also able to act as a downregulator of the classical pathway (11–14).

The coagulation pathway is another cascade-driven homeostatic system (15). The coagulation system, which is composed of cells, proteins, and processes that mediate blood clotting, is triggered by any damage to a blood vessel wall. This process is orchestrated through a series of proteolytic reactions (coagulation cascade), which are subdivided into the intrinsic, extrinsic, and common pathways (**Figure 1**). The extrinsic pathway is considered the first step in plasma-mediated hemostasis and is triggered by tissue factor (TF, or factor III), which is expressed in the subendothelial tissue, exposure to circulating factor VIIa, and calcium following vascular damage. The intrinsic pathway is initiated by the exposure of factor XII to subendothelial collagen, leading to a cascade of activations involving factors XI, IX, and VIII. Both pathways converge on the activation of factor X, signifying the commencement of the common pathway. Factor X, in conjunction with factor V, forms the prothrombinase complex, which catalyzes the conversion of prothrombin (factor II) into thrombin, the coagulation enzyme, which can convert soluble fibrinogen to insoluble fibrin (16, 17). The fibrinogen molecule is composed of two sets of three polypeptide chains termed α, β, and γ, which are held together by disulfide bonds (18). The homodimer (αβγ)_2_ is an elongated 45-nm structure consisting of two outer D domains, each connected to a central E domain by a coiled-coil segment. The E domain contains the fibrinopeptides A and B, while the γ′ segment contains a thrombin and FXIII binding site (19). The serine protease thrombin cleaves the amino-terminal regions of the α- and β-chains of fibrinogen and releases 2 mol each of fibrinopeptide A (FPA) and fibrinopeptide B (FPB for each mole of fibrin produced (20). Loss of FPA results in fibrin I, while additional loss of FPB yields fibrin II. The fibrin II monomers polymerize through end-to-middle domain (D:E) association to form double-stranded fibrils, which then associate laterally to form fibrin fibers (21). These fibrin fibers form a network, and the final fibrin solution is converted to a gel when at least 20% of fibrinogen is converted to fibrin (19). Fibrin stabilization is accomplished by the action of factor XIIIa (plasma transglutaminase), formed through the cleavage of soluble factor XIII by thrombin, which introduces numerous covalent cross-links between these fibrin molecules. The resulting cross-linked fibrin web is able to capture platelets and red blood cells, effectively sealing the wound and stemming plasma loss. In addition to its primary role of providing scaffolding for the transvascular thrombus, fibrin participates in other biological functions involving unique binding sites (21). These include i) the suppression of plasma factor XIIIa-mediated cross-linking activity in blood by binding factor XIIIa; ii) tissue type plasminogen activator (TPA) and plasminogen binding to fibrin, which results in the generation of plasmin, the major fibrinolytic protease (22); and iii) leukocyte binding to fibrin(ogen) via integrin α_M_β_2_ (Mac-1) (23).

Plasma FXIII is a tetrameric molecule consisting of two A- and two B-polypeptides held together non-covalently (24). The A-subunit contains the enzyme’s active site and is synthesized by hepatocytes and monocytes. It contains an activation peptide of 37 amino acids that limits the access of the substrate to the active-site cysteine. The B-polypeptide serves as a carrier for the hydrophobic A-subunit in human plasma, is synthesized by the liver, and is secreted as a monomer that binds free A in plasma (25). Plasma FXIII is converted to the active FXIIIa in two steps. In the first step, thrombin cleaves an activation peptide from the A-subunit with the formation of an inactive intermediate, FXIII′ (a′_2_b_2_) (26). A recent study has shown that thrombin hydrolysis of the plasma FXIII activation peptides is accelerated in the presence of fibrin I (27) through its interaction with the anion-binding exosite I (28) of FXIII. In the second step, calcium and fibrin induce the dissociation of the B-subunits from A to expose the active-site thiol group. Fibrin polymers are an important cofactor to generate FXIIIa. The generation of FXIIIa in plasma can be triggered when approximately 1%–2% of fibrinogen is converted to fibrin polymers (19). The activated FXIIIa first catalyzes the formation of γ-glutamyl/ε-lysyl bonds between fibrin γ-chains and then cross-links the α-chains of fibrin monomers (29). In addition to being a critical component of the coagulation system, FXIIIa also cross-links fibronectin, vitronectin, collagen, and lipoprotein in the extracellular matrix (30–33). Factor XIIIa also rapidly cross-links α2-antiplasmin (an inhibitor of plasmin) to the α-chain of fibrin (34), which inhibits the breakdown of fibrin by plasmin.

The complex interaction between the complement and coagulation systems is well established, primarily via *in vitro* studies (3). Briefly, the anaphylatoxins C3a and C5a promote both inflammation and coagulation by activating platelets and inducing their aggregation (35). Activated platelets, which are fundamental constituents of the coagulation cascade, are implicated in the initiation of both the classical and alternative pathways of the complement system (36–38). Factor H has been localized to the α-granules of platelets and is released in response to thrombin stimulation, or upon the binding of C3b to the platelet surface (39). Furthermore, factor H was found to co-purify from platelets with thrombospondin-1 (40). Factor H is also a substrate for FXIIIa (41–43). Thus, factor H present in platelets may participate in its interaction with the coagulation system, as platelets could release factor H at the site of clotting, while FXIIIa could anchor factor H at the site. Factor H also inhibits the activation of FXI by thrombin or FXIIa (44). FXIa can cleave factor H, reducing the binding of factor H to endothelial cells and, thereby, its activity in the factor I-mediated inactivation of C3b and C3b/Bb decay (45). C1 inhibitor is another complement regulatory protein that can influence the coagulation process through the inactivation of coagulation FXIIa (46). Similarly, MASP-2, a critical enzyme in the complement lectin pathway, can cleave thrombin directly from prothrombin (47). The terminal C5b-9 (MAC) also exhibits the ability to catalyze prothrombin activity, even in the absence of factor V (48). The cleaved complement component C5a, in particular, has been implicated in inducing procoagulant activity through a range of actions on endothelial cells and neutrophils, e.g., by inducing an increase in the expression of TF (49, 50) and instigating a switch in the activities of mast cells and basophils from a fibrin-dissolving (profibrinolytic) to a clot-forming (prothrombotic) role via the upregulation of plasminogen activator inhibitor-1 (PAI-1) (51). Similarly, thrombin promotes the direct activation of C3 and C5, independent of conventional complement pathway activation (52). Thrombin and plasmin can activate complement during liver regeneration, in a mouse model, in the absence of C4 and alternative pathway activity (53). Several coagulation factors, specifically FIXa, FXa, FXIa, and plasmin, can directly cleave C3 and C5 *in vitro*, resulting in the generation of the potent anaphylatoxins C3a and C5a (54–56). FXIIa can also stimulate the activation of the classical pathway via the C1 complex (57, 58).

In this study, we examined the involvement of factor H with the coagulation system. We characterized the interaction of factor H with fibrin clots immobilized on microtiter wells and those formed more physiologically in human plasma. The activation of the classical pathway by fibrin clots and the effects of factor H depletion on classical pathway activation were assessed.

## Materials and methods

### Preparation of fibrinogen- and fibrin-coated wells

To prepare fibrinogen-coated wells, 5 μg/well fibrinogen (100 μl/well of 50 μg/ml) was incubated with 2 mM iodoacetamide (IAM) and 1 mM Pefabloc-SC for 20 min at room temperature. The pre-incubated fibrinogen was then loaded onto microtiter plates (Maxisorp™) and the plates left for 1 h at 4°C. The plates were washed with phosphate-buffered saline (PBS)–0.5 mM EDTA and 0.1% Tween 20 and blocked with the same washing buffer for 2 h at room temperature. To coat the wells with fibrin, both fibrinogen (5 μg/well, 50 μl/well of 100 μg/ml; without IAM and Pefabloc-SC) and thrombin (12.5 ng/well, 50 μl/well of 0.25 μg/ml) were diluted in 20 mM HEPES, 120 mM NaCl, 5 mM CaCl_2_, and 0.05 mM DTT (pH 7.4). Fibrinogen was dispensed onto microtiter wells and left for 10 min at room temperature. Thereafter, thrombin was added to the wells and left for 40 min at 37°C. The plates were then transferred to 4°C, left for 20 min, and then washed and blocked similarly to the fibrinogen-coated plates.

### Binding assays of C1q or factor H to fibrin- or fibrinogen-coated wells

C1q and factor H were purified from pooled human serum as described before (59, 60) and were radioiodinated as previously described (61, 62). ^125^I-C1q or ^125^I-factor H was serially diluted starting at 500,000 cpm/well (125 and 160 ng/well for C1q and factor H, respectively) in a volume of 100 μl of 25 mM HEPES–0.1% Tween 20 (pH 7.4) or 100 μl of 25 mM HEPES–0.1% Tween 20 and 5 mg/ml bovine serum albumin (BSA) (pH 7.4). Dilutions were loaded onto fibrinogen- or fibrin- or BSA-coated wells and incubated for 30 min at 37°C. The wells were washed with HEPES buffer without BSA. To elute the bound ^125^I-C1q or ^125^I-factor H, 0.1 M NaOH was added to each well and allowed to incubate for 10 min. The supernatant was collected and counted in a Mini-Assay type 6–20 gamma counter (Mini-Instruments Ltd., Thermo Electronic Corporation, Reading, UK). This elution method was used in all subsequent plate-binding assays.

To study the saturation binding of ^125^I-factor H to fibrin, fibrin-coated wells were incubated with mixtures of a fixed amount of ^125^I-factor H (250,000 cpm, 80 ng/well) and various concentrations of unlabeled factor H (0–5,120 ng/well) for 30 min at 37°C. Bound ^125^I-factor H was eluted and quantified as described above. The total amount of factor H bound was calculated from the radioactivity bound per well and the known total amounts of factor H supplied.

To determine the inhibitory effects of an excess of unlabeled factor H on the binding of ^125^I-factor H to fibrin, ^125^I-factor H (2 μg/well) and different concentrations of unlabeled factor H (0–30 μg/well) or ovalbumin (0–9 μg/well) were premixed on ice and then incubated with fibrin-coated wells for 30 min at 37°C. Bound ^125^I-factor H was eluted and quantified as described above.

To study the effect of ionic strength on the binding of ^125^I-C1q or ^125^I-factor H (100,000 cpm) to fibrin, fibrin-coated wells were incubated with ^125^I-factor H or ^125^I-C1q in each of four different veronal buffers (2.5 mM sodium barbital, 0.15 mM CaCl_2_, and 0.5 mM MgCl_2_, pH 7.4) containing 20, 75, 150, or 500 mM NaCl for 30 min at 37°C. All washes were carried out at the same NaCl concentration used in the incubation. Bound ^125^I-factor H was eluted and quantified as described above.

### Binding assays of C1q or factor H to fibrin clots formed in human plasma

The binding of ^125^I-C1q and ^125^I-factor H to clots was examined at three different ionic strengths. Hence, the plasma dialysed in HEPES–saline (final concentration, 90%; 0.5 ml) was diluted with: a) 0.5 ml water (to give a final salt strength of approximately 70 mM); b) 0.5 ml HEPES–saline (to give approximately 140 mM); or c) 1,990 mM NaCl–10 mM HEPES–0.5 mM EDTA, pH 7.4 (to give approximately 1 M). This gives a final concentration of 45% plasma. A fixed amount of ^125^I-C1q or ^125^I-factor H (25,000 cpm) was premixed with each diluted plasma. CaCl_2_ (final concentration, 20 mM) was then added and the mixtures incubated for 40 min at 37°C. The clots were centrifuged (1,000 × *g*, 5 min) at 4°C and the supernatants removed to measure the radioactivity of unbound C1q or factor H. The clots were then washed three times with the appropriate reaction buffer and the supernatants removed. Radioactivity in the supernatants recovered from the clotting reaction and washing was measured. The ^125^I-C1q or ^125^I-factor H bound to fibrin clots was quantified by subtracting the cpm remaining in the supernatants of washes from the initial cpm in each reaction. In order to examine whether an increased clot size has an effect on the binding of C1q or factor H, the experiments were modified by adding an extra fibrinogen solution to a final concentration of 2 mg/ml in 100 μl of each reaction mixture. The clots formed in this manner were called “enhanced clots.” The binding assays with the extra fibrinogen added were carried out in the same manner as for the non-enhanced clots. It was found that a high, easily measurable binding for both C1q and factor H was achieved at 70 mM salt strength with additional fibrinogen in clots so that the enhanced clots formed at this salt strength were used in most experiments (unless indicated otherwise). Furthermore, in order to identify whether factor H and C1q bound directly to fibrin or to other proteins present in the plasma clots, it was necessary to examine the binding to the fibrin clots that were made only from fibrinogen by treating with thrombin (denoted as “fibrin-only clots”). Fibrin-only clots were made from the same quantity of fibrinogen as that present in the plasma for the “enhanced clots” in the presence of ^125^I-factor H or ^125^I-C1q.

To assess whether the binding of ^125^I-C1q or ^125^I-factor H to fibrin clots is covalent, a fibrin clot–urea washing assay was performed. ^125^I-C1q or ^125^I-factor H (25,000 cpm) was incubated in clotting plasma for eight time intervals in the range 0–960 min at 37°C. The clots were then washed three times vigorously with 500 μl of 10 mM HEPES, 70 mM NaCl, 0.5 mM EDTA, and 5 M urea (pH 7.4), and further unbound labeled protein was measured from the supernatants. ^125^I-C1q or ^125^I-factor H, which remained associated with clots, was calculated as before. The remaining bound radioactive material was judged likely to be covalently bound. To investigate whether plasma proteins other than C1q and factor H interacted with fibrin clots, various radiolabeled plasma proteins were incubated in clotting plasma. The proteins used were ^125^I-C1q, ^125^I-factor H, ^125^I-human serum albumin (HSA), ^125^I-plasminogen, ^125^I-transferrin, ^125^I-IgG, and ^125^I-α-2-macroglobulin (25,000 cpm for each protein). HSA, plasminogen, IgG, and α-2-macroglobulin were purified at the MRC Immunochemistry Unit, Oxford, and iodinated as described previously (61). Enhanced fibrin clots were formed in the presence of the plasma proteins. After 16 h incubation, the fibrin clots were washed three times in 500 μl of 10 mM HEPES, 70 mM NaCl, 0.5 mM EDTA, and 5 M urea (pH 7.4). Unbound labeled proteins were measured from the supernatants, and the labeled protein that remained associated with clots was calculated as follows: radioactivity in the supernatants recovered from the clotting reaction and washing was measured. The ^125^I-C1q or ^125^I-factor H, bound to fibrin clots, was quantified by subtracting the cpm remaining in the supernatants of washes from the initial cpm in each reaction. In order to determine whether C1q or factor H was covalently linked to fibrin clots by the action of FXIIIa, ^125^I-C1q or ^125^I-factor H (25,000 cpm) was premixed with 10-fold diluted plasma (0.9%, final concentration in HEPES–1/2-saline) and 20 μg of fibrinogen in a total volume of 50 μl of HEPES–1/2-saline. CaCl_2_ was added to a final concentration of 20 mM, and the mixture was incubated for 16 h at 37°C. As controls, either 20 mM ϵ-amino caproic acid (EACA; final concentration) was added to the mixtures before the addition of CaCl_2_ and pre-incubated for 5 min at room temperature, or 5 mM IAM (final concentration) was added at the same stage for 10 min at room temperature. EACA and IAM were used as negative controls as they inhibit FXIIIa. The clots were washed three times with HEPES–1/2-saline or with 10 mM HEPES, 70 mM NaCl, 0.5 mM EDTA, and 5 M urea (pH 7.4). The clots were then suspended in SDS-PAGE sample buffer containing 50 mM DTT and further analyzed with SDS-PAGE (4%–12% acrylamide) under reducing conditions.

### Hemolytic complement assay: measurement of C4 consumption (via the classical pathway)

Sensitized sheep erythrocytes (EA) for the C4 consumption assay were prepared as described previously (9). Factor H-depleted plasma was prepared from normal human plasma as described previously (63). In an ELISA, factor H-depleted plasma was then verified as being approximately 99.9% factor H-deficient compared to normal plasma (data not shown). C4 consumption by clot formation in normal human plasma and factor H-depleted plasma was determined via a hemolytic assay. Firstly, C4-deficient guinea pig serum (Sigma-Aldrich, St. Louis, USA) was titrated on EA cells to determine a suitable dilution, which did not cause complement-mediated lysis of the cells. Thus, C4-deficient guinea pig serum was serially diluted from 1/2 to 1/1,024 in a volume of 100 μl with DGVB^2+^ in microtiter plates, and then each dilution was incubated with 100 μl of EA cells in DGVB^2+^ (1 × 10^8^ cells/ml) for 1 h at 37°C. The cells were spun down at 1,000 × *g* for 10 min and the optical density of the supernatants measured at 414 nm. A positive control for 100% EA lysis was achieved through lysis of the erythrocytes with H_2_O. The negative control of DGVB^2+^ added to the EA cells gave the 0% lysis baseline. The background 0% lysis was subtracted from each supernatant OD_414_ reading, and then the hemolytic activity was measured relative to 100% lysis-positive control. As a result, 1/32 dilution of the C4-deficient guinea pig serum was selected for assays to determine the changes in the C4 levels (C4 consumption) by clots. Secondly, C4 consumption by fibrin clots was measured in normal human plasma (1/10 dilution in HEPES–1/2-saline) or in factor H-depleted plasma (1/10 dilution in HEPES–1/2-saline) supplemented with fibrinogen (2 mg/ml) and was allowed to clot in a total volume of 100 μl of HEPES–1/2-saline. Clotting was initiated by adding CaCl_2_ to 20 mM and the samples incubated for 16 h at 37°C. The samples were centrifuged at 10,000 × *g* for 10 min at 4°C and the supernatant removed and further diluted 10-fold in DGVB^2+^. This diluted supernatant (100 μl) was then loaded into microtiter plates and serially diluted with DGVB^2+^ from 1/2 to 1/4,096 (equivalent to final dilutions of the plasma from 1/200 to 1/409,600). Each dilution was mixed with a fixed concentration of guinea pig serum (100 μl of 1/32 dilution in DGVB^2+^) on ice. EA cells (10^7^ cells/well in DGVB^2+^) were placed in microtiter plates, centrifuged for 10 min at 1,000 × *g*, and the supernatants were removed. The assay mixtures were then transferred to the wells containing EA cells and incubated for 1 h at 37°C. The cells were spun down at 1,000 × *g* for 10 min and the OD_414_ of the supernatant was read. The percentage lysis was calculated as described above. The activity of C4 was expressed as the reciprocal dilution of serum/plasma required to give 50% lysis, which was expressed as units of C4 activity per unit volume. The amounts of C4 consumption by clots were calculated by comparing with unclotted plasma. This assay measured the extent of C4 consumption (reduction in the level of C4) that occurred during clotting in the normal or factor H-depleted plasmas. Further control experiments were carried out, as above, with normal plasma and factor H-depleted plasma, but in the absence of clotting.

### Hemolytic complement assay: measurement of MAC formation

MAC formation arising from complement activation by fibrin clots was determined using an ELISA system. MAC deposition was measured on fibrin-coated wells, while MAC formation was also measured in the supernatants recovered from fibrin-coated wells incubated with serial dilutions of human serum. Ovalbumin antigen–antibody (OVA ag–ab) complexes and ovalbumin were used as a positive and a negative control, respectively. Firstly, fibrin- or fibrinogen-coated (see above for preparation) or OVA ag–ab complex- or ovalbumin-coated plates were prepared. OVA ag–ab complexes were prepared on plates as follows: hen ovalbumin (antigen) was dissolved in 0.1 M sodium carbonate (pH 9.6). Ovalbumin (100 μl of 50 μg/ml) was then added to each well and incubated for 1 h at room temperature. The plates were blocked with PBS–0.5 mM EDTA and 0.1% Tween 20 for 2 h at room temperature. Each well was then incubated with 200 μl of a 1:1 dilution of rabbit anti-ovalbumin antiserum (MRC Immunochemistry Unit, Oxford, UK) in 1.5 M NaCl and 50 mM EDTA (pH 7.4) for 1 h at room temperature. The plates were washed with 750 mM NaCl and 25 mM EDTA (pH 7.4), and then washed again with PBS–0.5 mM EDTA and 0.1% Tween 20. This gives OVA ag–ab complexes with some IgM, but mostly IgG. The high NaCl concentration prevents the binding of rabbit C1 or C1q. Secondly, fresh human serum was serially diluted (from 1/10 to 1/390,6250) in a total volume of 100 μl of DGVB^2+^ buffer on ice, immediately transferred to the fibrin-, fibrinogen-, ovalbumin-, and OVA ag–ab complex-coated wells, and then incubated for 1 h at 37°C. After incubation, the plates were centrifuged at 1,000 × *g* for 10 min and the supernatants were removed and kept at −20°C. The wells were washed with PBS–0.5 mM EDTA and 0.1% Tween 20 and incubated with monoclonal anti-neo C9 antibody (3.75 μg/ml) for 1 h at room temperature. The mouse anti-neo C9 antibody (750 μg/ml in PBS–0.1% sodium azide) was a kind gift from Prof. Reinhard Wurzner (Innsbruck Medical University, Innsbruck, Austria). It detects a neoepitope that is formed on C9 when incorporated into MAC. The wells were washed with PBS–0.5 mM EDTA and 0.1% Tween 20 and then incubated with 100 μl of a goat anti-mouse IgG alkaline phosphatase conjugate (1/5,000 dilution in PBS–0.5 mM EDTA; Sigma) for 1 h room temperature. The wells were washed again in PBS–0.5 mM EDTA and 0.1% Tween 20 and visualized using the soluble alkaline phosphatase substrate *p*-nitrophenyl phosphate. A substrate solution (100 μl) was added to each well, incubated at room temperature in the dark, and the OD_405_ was read. Serial dilutions of fresh human serum were also incubated with blocked wells only to detect the nonspecific formation of MAC in the human serum; the values for these wells were subtracted from the values of each sample. Furthermore, MAC was also measured in the supernatants of serum incubated in wells using a capture ELISA system. In order to capture MAC in the supernatants, a capture antibody (rabbit anti-human C9 IgG) was purified from rabbit anti-human C9 antiserum using a HiTrap Protein G column (Sigma-Aldrich). The plates were coated with rabbit anti-human C9 IgG (5 μg/well in 0.1 M sodium carbonate, pH 9.6) for 1 h at 20°C. After incubation, the wells were washed with PBS–0.5 mM EDTA and 0.1% Tween 20 and blocked in the same buffer for 2 h at 20°C. The supernatants of the serially diluted human serum removed from each fibrin-, fibrinogen, OVA ag–ab complex-, and ovalbumin-coated wells were then added to the wells and incubated for 1 h at room temperature. The wells were washed and MAC was detected using the anti-neo C9 antibody, as described above. The supernatants of the serially diluted fresh human serum recovered from blocked wells only were also assayed in the capture ELISA to detect background MAC formation in the human serum. Background values were subtracted from the values of each sample.

### Binding of ^125^I-C1q or ^125^I-factor H to “enhanced clots” formed in the presence or absence of plasma

Enhanced plasma clots were formed in the presence of ^125^I-C1q or ^125^I-factor H (25,000 cpm), as described above. The clots were washed with 500 μl of HEPES–1/2-saline buffer or with 10 mM HEPES, 70 mM NaCl, 0.5 mM EDTA, and 5 M urea (pH 7.4). The concentration of fibrinogen in the “enhanced clots” mixture was 3.5 mg/ml. Thus, to produce clots in the absence of plasma, 3.5 mg/ml purified fibrinogen in HEPES–1/2-saline buffer was used. The fibrinogen solution (10 ml of 35 μg/ml) was premixed with ^125^I-C1q or ^125^I-factor H (25,000 cpm) in a total volume of 96.5 μl in 10 mM HEPES, 70 mM NaCl, and 5 mM CaCl_2_ (pH 7.4). Clotting was initiated by adding 3.5 μl thrombin (final concentration, 8.75 μg/ml). This quantity of thrombin was selected as the incubation of fibrinogen with thrombin at the same weight ratio resulted in complete cleavage of the α- and β-chains of fibrinogen at 40 min incubation at 37°C, as assessed in our previous experiment (data not shown). As for the enhanced clots, the mixture was incubated for 40 min at 37°C and the resulting clots processed and washed similarly to the “enhanced clots.” The ^125^I-C1q or ^125^I-factor H that remained associated with clots was measured as above.

### ELISA comparison of C1q or factor H in human plasmas and sera

C1q or factor H was detected from five different samples of citrated plasma and the serum from the same plasmas. These plasmas represented a range of age in terms of storage and condition, which included human normal plasma citrated (HPNC); platelet-poor plasma (PPP thawed); pooled citrated fresh plasma from a healthy volunteer (FHV); and O Rhesus positive. HPNC, PPP thawed, and citrated FHV were all pooled outdated human plasma from HDS Supplies. O Rhesus positive was obtained from an 8-year-old sample kept at −20°C. In order to detect C1q, OVA ag–ab complex-coated wells were used (see above for description). Serial twofold dilutions of each plasma and serum pair from 1/10 to 1/5,120 in PBS–0.1% Tween 20–5 mM EDTA were incubated with the OVA ag–ab-coated wells for 1 h at room temperature. The wells were washed with PBS–0.1% Tween 20 and then incubated with 100 μl of 100 μg/well biotinylated rabbit anti-human C1q (Sigma) for 1 h at room temperature. The wells were washed and incubated with 100 μl/well of 1/40,000 extravidin–alkaline phosphatase (Sigma) for 30 min at 20°C. The wells were washed, the plate was developed with *p*-nitrophenyl phosphate, and the OD was read at 405 nm. Factor H was detected in the plasma and serum pairs as follows: wells coated with the monoclonal anti-human factor H MRCOX23 (2.5 μg/well of purified IgG; MRC Immunochemistry Unit) were used to capture factor H. Serial twofold dilutions of each plasma and serum made from 1/20 to 1/10,240 in PBS–0.1% Tween 20 were incubated with the anti-factor H-coated wells for 1 h at 20°C. The plates were washed with PBS–0.1% Tween 20 and further incubated with rabbit anti-human factor H (MRC Immunochemistry Unit) at a dilution of 1/32,000 in PBS–0.1% Tween 20 for 1 h at 20°C. The wells were incubated with 100 μl of 1/10,000 dilution of a goat anti-rabbit–IgG alkaline phosphatase conjugate (Sigma) for 1 h at 20°C. After incubation, the wells were washed and visualized using an alkaline phosphatase substrate, *p*-nitrophenyl phosphate (Sigma). The readings were taken at OD_405_.

### Statistical analysis

Statistical significance was considered as indicated in the figure legends. Error bars show the SD or SEM, as stated in the figure legends.

## Results

### Factor H and C1q interact with fibrin in the absence of BSA

**Figure 2:**
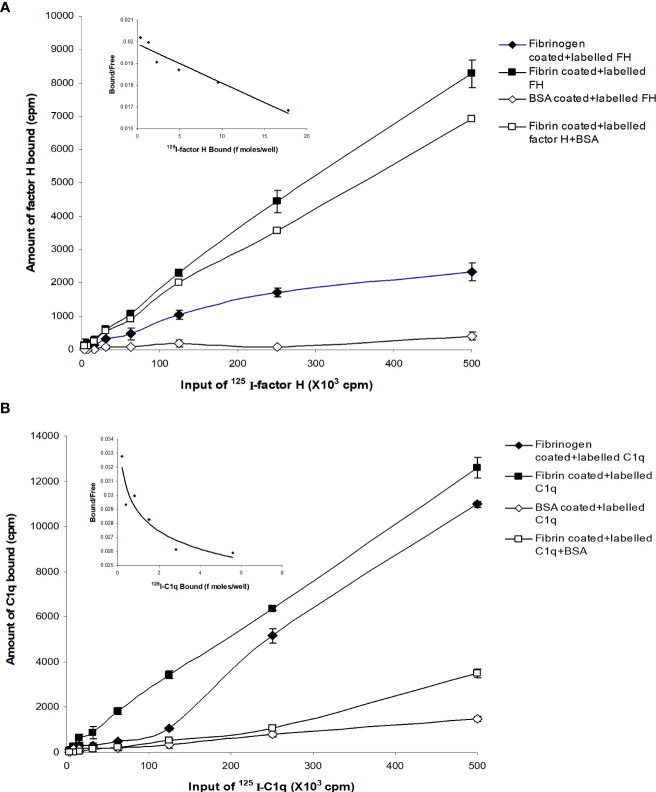
Binding of ^125^I-factor H, and ^125^I-C1q to fibrinogen-, fibrin-, and bovine serum albumin (BSA)-coated wells. A set of two-fold serial dilutions of ^125^I-factor H **(A)** or ^125^I-C1q **(B)** (starting at 500,000 cpm/well of ^125^I-factor H or ^125^I-C1q) was incubated with fibrinogen or fibrin for 30 min at 37°C in 20 mM HEPES–0.1% Tween 20 (pH 7.4). The microtitre wells were washed and the amounts of bound factor H and C1q were measured. BSA-coated wells were used as a negative control. Simultaneously, experiments were carried out with the sample dilutions of ^125^I-factor H or ^125^I-C1q in 20 mM HEPES–0.1% Tween 20 (pH 7.4) containing 5 mg/ml BSA. All experiments were performed in triplicate (*n* = 3) and the average of two independent experiments (*N* = 2). A Scatchard plot of bound/free *versus* bound for each ^125^I-factor H or ^125^I-C1q binding to fibrin-coated wells are shown (*top*, *left panel*). The linear plot for factor H binding indicates that the fibrin clots have one receptor class (a single affinity receptor) with the slope of the line equal to −1/*K*_d_. The intercept on the *X*-axis estimates the total ligand bound if all the receptor sites were occupied.

Different concentrations of ^125^I-factor H or ^125^I-C1q were allowed to bind to fibrinogen-, fibrin-, or BSA-coated microtiter wells. The dose-dependent binding of ^125^I-factor H (**Figure 2A**) or ^125^I-C1q (**Figure 2B**) to fibrinogen or fibrin was observed. ^125^I-factor H and ^125^I-C1q both showed higher binding to fibrin than to fibrinogen, although for C1q, the difference between fibrin and fibrinogen binding was small. When ^125^I-factor H (maximum quantity, 500,000 cpm/well) was added to fibrin-coated wells, approximately 1.7% of factor H was bound. For ^125^I-C1q (500,000 cpm/well), approximately 2.4% was bound to fibrin-coated wells. The binding of both ^125^I-factor H and ^125^I-C1q to fibrin was therefore low. The binding of ^125^I-C1q to fibrinogen- and fibrin-coated wells (only the data for fibrin are shown) was greatly decreased in the presence of fluid-phase BSA, whereas the binding of ^125^I-factor H was only slightly diminished by BSA (**Figure 2**). ^125^I-factor H, therefore, showed binding to fibrin that was specific in that it was not competed out by BSA. The amount of factor H bound was low, but it was considered of sufficient interest to merit further investigation of the binding characteristics of ^125^I-factor H to fibrin. The binding of ^125^I-factor H and ^125^I-C1q to BSA-coated wells was very low. To determine the affinity of ligand binding to macromolecules (fibrin) and the number of receptors per fibrin-coated well, Scatchard plots were drawn, where the *X*-axis represents specific binding (B) and the *Y*-axis denotes specific binding divided by the free radioligand concentration (B/F), as shown in **Figure 2** (top, left panel). The Scatchard plot for factor H binding was linear, suggesting that there was one class of receptors. The *K*_d_ (dissociation constant) indicates the strength of binding (affinity) between the receptors and their ligands. If the *K*_d_ is low, the affinity is high. For factor H, the *K*_d_ was 55.0 pM (slope equals −1/*K*_d_, where slope was −1.82 × 10^−3^ fmol^−1^). This *K*_d_ value indicates an exceptionally high affinity. The *X*-intercept was 20.5 fmol, which is the total ligand bound in femtomoles per well. Thus, each fibrin-coated well was able to bind to a maximum number of 1.234 × 10^10^ factor H molecules (maximum number of ligand moles bound × Avogadro’s constant). Thus, there are 1.234 × 10^10^ factor H binding sites per fibrin-coated well. Therefore, the affinity of factor H for fibrin is high, but the number of ligand sites is low. The Scatchard plot representing the binding of ^125^I-C1q was nonlinear, which is consistent with the multivalency of C1q.

**Figure 3:**
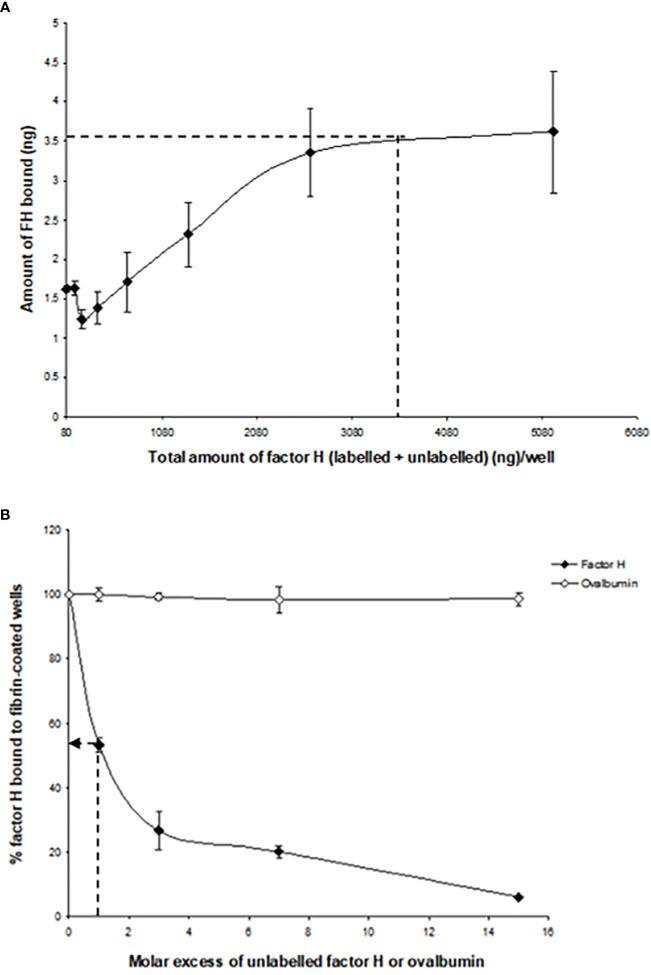
Characterization of the binding of ^125^I-factor H to fibrin-coated wells. **(A)** Saturation of the binding of ^125^I-factor H to fibrin-coated wells. Different amounts of unlabeled factor H (0–5,120 ng/well) were mixed with a fixed amount of ^125^I-factor H (250,000 cpm/well, equivalent to 80 ng/well) on ice and loaded onto fibrin-coated wells. Saturation was observed at an input of approximately 3.5 μg of factor H per well (denoted by a *dotted mark*). The means of three experiments are presented with standard deviations. **(B)** Inhibition of the binding of ^125^I-factor H to fibrin by excess factor (H) Labeled factor H (2 μg/well) was incubated with increasing amounts of unlabeled factor H (0–30 μg/well) in fibrin-coated wells for 30 min at 37°C. The wells were washed and the amount of ^125^I-factor H bound was measured as cpm bound per well. Ovalbumin (0–9 mg/well) was used as a control. The *dotted arrow* represents the percentage of factor H bound to fibrin when a 1:1 molar ratio of factor H to C1q was used. The means of three independent experiments performed in triplicate (*N* = 3; *n* = 3) are presented with standard deviations.

The binding of factor H to fibrin was dose-dependent and saturable. Saturation was achieved at an input of approximately 3.5 μg of factor H (**Figure 3A**). Approximately 3.5 ng of factor H was bound to fibrin-coated wells with an input of 3.5 μg; thus, 0.1% of binding was achieved. To determine the inhibitory effects of excess of unlabeled factor H on the binding of ^125^I-factor H to fibrin, both labeled (2 μg) and unlabeled factor H (0–30 μg/well) were premixed on ice and then incubated with fibrin-coated wells. The results showed that there was a decrease in the binding of ^125^I-factor H to fibrin-coated wells as the concentration of unlabeled factor H increased (**Figure 3B**). At an equal molar ratio of unlabeled to labeled ligand, unlabeled factor H inhibited the binding of ^125^I-factor H to fibrin-coated wells by 48%. Therefore, unlabeled factor H predictably inhibits the binding of labeled factor H. Ovalbumin did not interfere with the binding of ^125^I-factor H to fibrin (**Figure 3B**).

**Figure 4:**
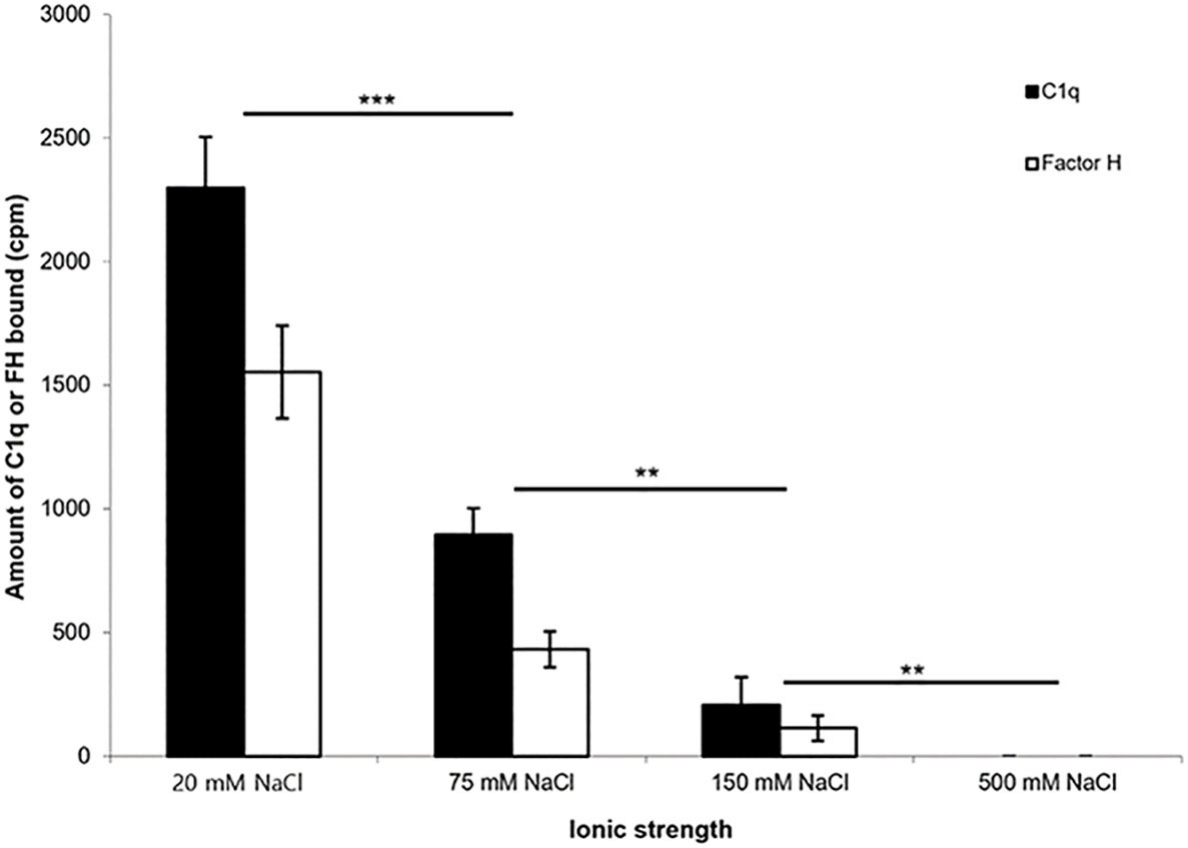
Effect of ionic strength on the binding of ^125^I-factor H or ^125^I-C1q to fibrin. ^125^I-factor H (32 ng, 100,000 cpm) or ^125^I-C1q (20 ng, 100,000 cpm) at four different ionic strengths (20, 75, 150, and 500 mM NaCl) in VB^2+^ buffer was incubated with fibrin-coated wells. Three independent experiments were conducted in triplicate. *Error bars* represent ±standard deviations. Significance was determined using unpaired *t*-test. **p < 0.01, ***p < 0.001 (*N* = 3; *n* = 3).

To determine whether the interaction of ^125^I-factor H or ^125^I-C1q with fibrin-coated wells is affected by ionic strength, ^125^I-factor H or ^125^I-C1q in veronal buffer with four different ionic strengths (20, 75, 150, and 500 mM NaCl) was incubated in fibrin-coated wells. Very high binding of ^125^I-factor H or ^125^I-C1q was observed at 20 mM NaCl (**Figure 4**). The binding at 75 mM NaCl concentration was less than 50% of that seen at 20 mM NaCl. The binding of ^125^I-factor H and ^125^I-C1q was greatly decreased at 150 mM NaCl. At 500 mM NaCl, binding was not detectable for either ^125^I-factor H or ^125^I-C1q. This indicates that the binding of ^125^I-factor H or ^125^I-C1q is ionic in nature. Furthermore, the binding of both factor H and C1q was independent of divalent metal ions, as a similar binding was observed with HEPES–saline–EDTA (5 mM EDTA was added to HEPES–saline) and HEPES–1/2-saline (data not shown).

### ^125^I-factor H and ^125^I-C1q interact with clots formed in plasma

Factor H and probably C1q appear to bind to fibrin-coated wells, as determined by the plate assays (**Figures 2**–**4**). It was therefore of interest to examine whether an interaction of ^125^I-factor H or ^125^I-C1q with fibrin clots can be measured in more physiological conditions. The coating of plates with the fibrin/fibrinogen used in this work is quite artificial as the plate-fixed fibrin cannot move to form a fibrin polymer, as occurs in clots. To simulate real conditions, fibrin clots were formed in plasma in the presence of ^125^I-factor H or ^125^I-C1q. Before studying the binding of factor H or C1q to fibrin clots, the quantity of ^125^I-fibrinogen incorporated into cross-linked fibrin clots was determined (data not shown). In addition, the efficiency of washing of fibrin clots was examined. Approximately 89% of ^125^I-fibrinogen was incorporated into clots. Unbound ^125^I-fibrinogen or fibrin (approximately 8.5%) was removed mostly in the first supernatant of the clotting reaction, and only a small amount of ^125^I-fibrinogen or fibrin (<1%) was detected in all three washes. Unbound ^125^I-fibrinogen could occur because not all of the fibrinogen is activated by thrombin, or not all of the fibrin is cross-linked by FXIIIa.

**Figure 5:**
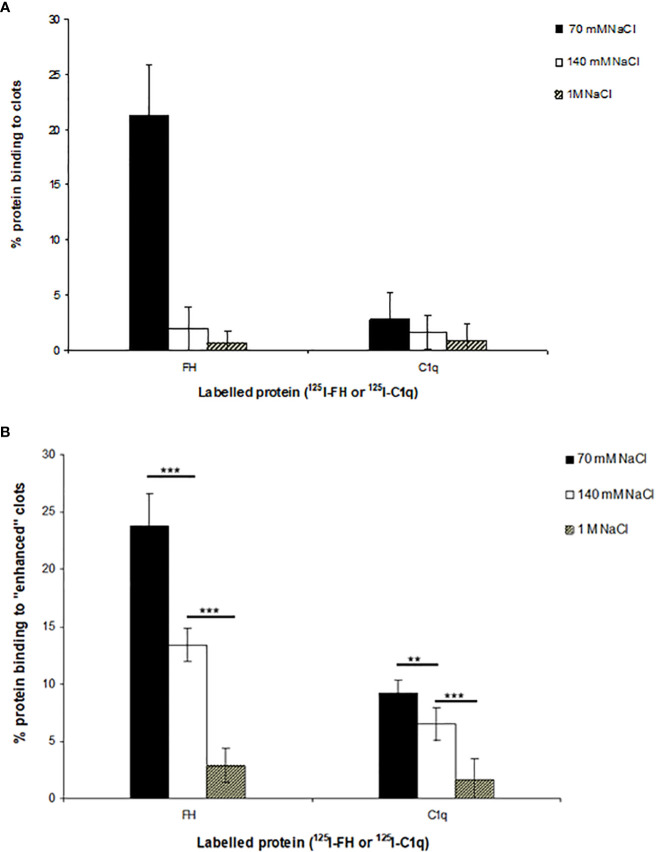
Interaction of ^125^I-factor H or ^125^I-C1q with the plasma clots formed in more physiological conditions. **(A)** Clots were formed in plasma in the presence of ^125^I-factor H (5 ng, 25,000 cpm/reaction) or ^125^I-C1q (8 ng, 25,000 cpm/reaction) at three salt strengths (70 mM, 140 mM, and 1 M NaCl). **(B)** Enhanced clots were also formed in the presence of ^125^I-factor H or ^125^I-C1q (25,000 cpm/reaction) at different ionic concentrations (70 mM, 140 mM, and 1 M NaCl). The means of three experiments are presented with standard deviations. Three independent experiments were conducted in triplicate. *Error bars* represent ±standard deviations. Significance was determined using unpaired *t*-test. **p < 0.01, ***p < 0.001 (*N* = 3; *n* = 3).

The binding of ^125^I-factor H and ^125^I-C1q to clots was examined at three different ionic strengths. Clots were formed in human plasma in the presence of ^125^I-factor H or ^125^I-C1q at three different salt concentrations, and the percentage of ^125^I-factor H or ^125^I-C1q associated with the clot was determined by measuring the depletion of radioactivity from the supernatant after clot formation. The binding rates of ^125^I-factor H and ^125^I-C1q were 21.5% and 2.5%, respectively, at 70 mM salt concentration (**Figure 5**). There was also some detectable binding at 1 M NaCl. The percentage binding of both factor H and C1q to these fibrin clots was much greater than that observed in the plate assays. In order to optimize clot formation and the potential binding to clots by ^125^I-factor H or ^125^I-C1q, fibrinogen (final concentration, 2 mg/ml) was added to the reaction mixture prior to clot formation. This produced “enhanced” fibrin clots. In comparison to the binding shown in **Figure 2**, there was an “enhanced” binding of ^125^I-factor H and ^125^I-C1q observed at various salt concentrations when fibrinogen was added in order to increase the clot size (**Figure 5B**). The percentage of bound ^125^I-factor H was increased six-fold at a physiological salt concentration (140 mM NaCl), but no significant increase in binding was observed at 70 mM salt concentration. The binding of ^125^I-C1q was enhanced compared to normal clots at all salt concentrations. Therefore, the binding of ^125^I-factor H and ^125^I-C1q to fibrin was greater when the clot volume/surface area was increased by adding more fibrinogen into the clotting mixture. This was to increase the concentration of epitopes for the binding of factor H or C1q. Although increasing the salt strength may have altered the kinetics of clot formation, clot formation was observed by eye at all the strengths tested.

### Evidence for the covalent binding of ^125^I-factor H and ^125^I-C1q to enhanced fibrin clots

**Figure 6:**
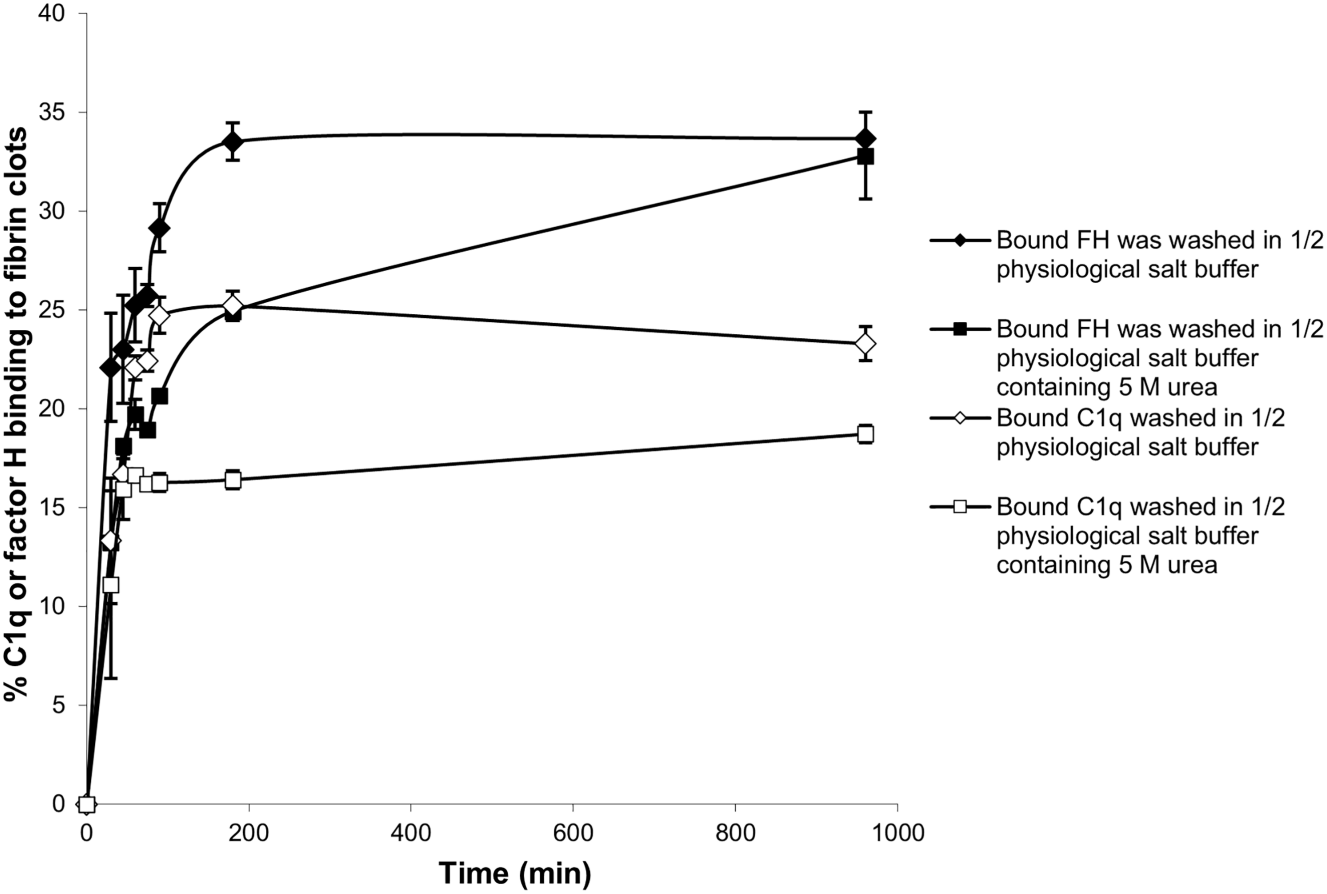
Time course of the binding of ^125^I-factor H and ^125^I-C1q to fibrin clots. Enhanced fibrin clots were formed in the presence of ^125^I-factor H or ^125^I-C1q, but the clotting mixtures were incubated for eight time intervals in the range 0–960 min at 37°C. The enhanced clots were washed with HEPES–1/2-saline or in 10 HEPES, 70 mM NaCl, 0.5 mM EDTA, and 5 M urea (pH 7.4). The means of three experiments performed in triplicate and standard deviations are plotted.

Further experiments were carried out with the enhanced fibrin clots. To determine whether the binding of ^125^I-factor H and ^125^I-C1q to fibrin clots is covalent, i.e., involving cross-linking by FXIIIa, a kinetic experiment of the binding of ^125^I-factor H or ^125^I-C1q to fibrin clots was performed. After incubation for various time points, fibrin clots with bound ^125^I-factor H or ^125^I-C1q were washed with 10 mM HEPES, 70 mM NaCl, 0.5 mM EDTA, and 5 M urea (pH 7.4) in order to denature the non-covalently bound proteins. The proportions of ^125^I-factor H and ^125^I-C1q binding to clots increased as the incubation time increased (**Figure 6**). The binding of both ^125^I-factor H and ^125^I-C1q reached its maximum level after 100–180 min incubation, as assessed after washing with HEPES–1/2-saline. Most of the ^125^I-factor H and ^125^I-C1q remained in the fibrin clots after the urea wash. Thus, it is likely that both proteins are covalently bound to fibrin. The covalent association of ^125^I-factor H and ^125^I-C1q still increased gradually with incubation beyond 180 min. When the fibrin clots were washed with urea, the binding of ^125^I-factor H and ^125^I-C1q was reduced. For example, the proportions of the binding of ^125^I-factor H and ^125^I-C1q were reduced from 22% to 13% and from 13% to 11%, respectively, at 30 min incubation. At 96 min incubation, 26% of factor H was shown to bind to fibrin clots after washing in non-denaturing buffer, but only 18% remained bound after washing in urea buffer. Thus, non-covalently bound factor H or C1q was easily dissociated by urea. After 960 min incubation, approximately 32.5% of ^125^I-factor H and 17.6% of ^125^I-C1q remained bound to fibrin clots after washing in urea. The covalent binding between factor H and fibrin clots appeared to be complete at 960 min.

### Plasminogen interacts covalently with enhanced fibrin clots, but HSA, transferrin, IgG, and α-2-macroglobulin do not

Factor H and C1q are large asymmetric proteins that could possibly be trapped in the clots as fibrin cross-linking increases. To determine whether the interaction of C1q and factor H with fibrin clots is not simply by physical entanglement, binding assays were carried out using other plasma proteins with various molecular weights, which included HSA (66 kDa), plasminogen (90 kDa), transferrin (81 kDa), IgG (150 kDa), and α-2-macroglobulin (720 kDa), as well as factor H (155 kDa) and C1q (410 kDa). These proteins were radioiodinated and added to human plasma, which was supplemented with 2 mg/ml fibrinogen (final concentration). This formed enhanced fibrin clots by adding CaCl_2_ (final concentration, 20 mM) and incubating for 16 h at 37°C. The enhanced fibrin clots were washed three times in urea buffer, and the amount of proteins bound to the fibrin clots was measured (**Table 1**). There was significant binding of C1q (28.5%), factor H (30%), and plasminogen (31%) to fibrin clots when 25,000 cpm was added to each reaction (0.5 nM factor H, 0.1 nM C1q, and 1.4 nM plasminogen). The other plasma proteins (i.e., HSA, transferrin, IgG, and α-2-macroglobulin) showed low binding (<5%), which could be the result of some traces of simple physical entrapment in the clots. Therefore, factor H and C1q are retained in fibrin clots, not by physical entanglement but by specific binding followed by covalent cross-linking. Plasminogen was likely to be covalently bound to fibrin clots as the binding was stable after washing with urea.

**Table 1:** Interaction of plasma proteins with fibrin clots.

	Protein incorporated into fibrin clots (% ± SD)
C1q	30.2 ± 2.6
Factor H	28.7 ± 4.8
Human serum albumin	2.1 ± 1.0
Plasminogen	32.8 ± 5.6
Transferrin	1.7 ± 0.5
IgG	1.8 ± 0.4
α-2-Macroglobulin	2.3 ± 1.1

To determine whether the interaction of factor H and C1q with fibrin clots is not simply due to physical entanglement, a binding assay was carried out using other plasma proteins. Enhanced fibrin clots were formed in the presence of various radiolabeled plasma proteins including ^125^I-factor H (5 ng), ^125^I-C1q (6 ng), ^125^I-human serum albumin (11 ng), ^125^I-plasminogen (12.5 ng), ^125^I-transferrin (20 ng), ^125^I-IgG (6.25 ng), and ^125^I-α-2-macroglobulin (16 ng) (25,000 cpm/reaction used for each protein). The clotting reaction was allowed to proceed for 16 h at 37°C. After incubation, fibrin clots were washed three times in 10 mM HEPES, 70 mM NaCl, 0.5 mM EDTA, and 5 M urea (pH 7.4). The amounts of plasma proteins bound to the fibrin clots were measured, as described in Materials and methods. The means of three experiments are presented with standard deviations.

### Evidence for the covalent binding of factor H and C1q to fibrin clots via FXIIIa (transglutaminase)

**Figure 7:**
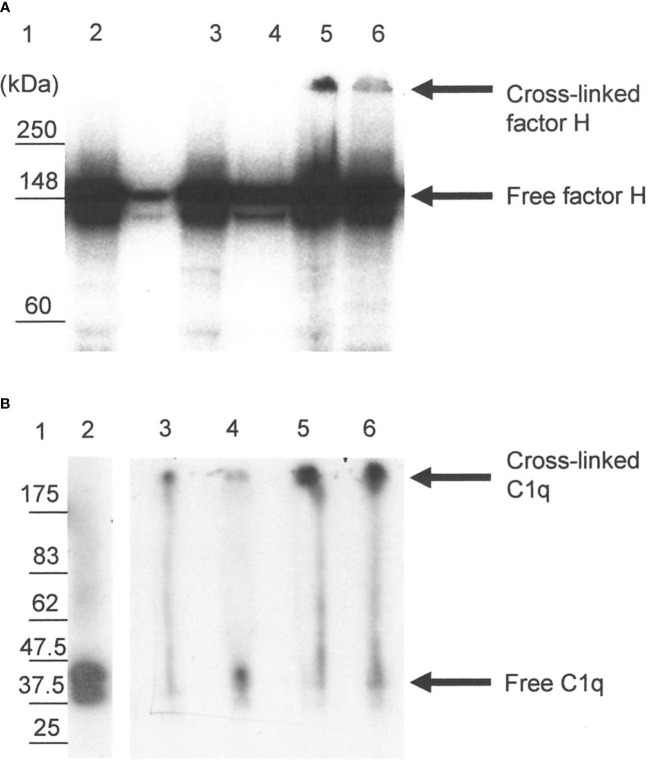
SDS-PAGE analysis of the ^125^I-factor H **(A)** and ^125^I-C1q **(B)** cross-linked to clots. Clots were formed in human plasma in the presence of ^125^I-factor H or ^125^I-C1q or ^125^I-plasminogen, and the remaining bound materials were examined by SDS-PAGE (4%–12% gradient gel) under reducing conditions. SDS-PAGE gels containing ^125^I-labeled materials were dried and exposed to an X-ray film in autoradiography cassettes, and the films were developed on a Kodak X-OMAT processor model ME-1. Various possible inhibitors of cross-linking, e.g., epsilon amino caproic acid (EACA) and iodoacetamide (IAM), were incubated with plasma prior to the addition of CaCl_2_. *Lane 1*, standard protein molecular mass marker; *lane* 2, standard ^125^I-factor H or ^125^I-C1q; *lane 3*, incubation with EACA before Ca^2+^; *lane 4*, addition of IAM before Ca^2+^; *lane 5*, ^125^I-factor H or ^125^I-C1q bound to clots without inhibitors; *lane 6*, ^125^I-factor H or ^125^I-C1q from the supernatant of the clotting mixture. It is likely that the ^125^I-factor H and ^125^I-C1q bound to clots show up as a band corresponding to the cross-linked products that remained in the well (*lane 5*). No cross-linked products were observed with inhibitor controls (*lanes* 3 and *4*). A representative blot of three independent replicates is presented.

The reaction of ^125^I-factor H and ^125^I-C1q with fibrin clots was examined using SDS-PAGE. On a 4%–12% SDS-PAGE under reducing conditions, 5.2% of ^125^I-factor H (assessed by scanning the autoradiograph and analyzing using Image Gauge software, Fuji FLA 3000 Imager) was observed as a large aggregate that did not migrate into the gel (**Figure 7A**, lane 5). This suggests that ^125^I-factor H was bound to the fibrin clots covalently. There was also a small proportion of cross-linked products in the supernatant removed from the clots (**Figure 7A**, lane 6). When fibrin clots were formed with ^125^I-C1q and washed with urea, ^125^I-C1q was also found to be linked to the clots (**Figure 7B**, lane 5). Therefore, ^125^I-C1q was also covalently bound to fibrin clots. It was of interest to determine whether this covalent binding involves cross-linking by FXIIIa. In order to investigate whether factor H and C1q are a target for FXIIIa, EACA or IAM was used as an inhibitor. EACA, a competitive inhibitor for COOH and NH_2_ donor and acceptor groups, competes with fibrin for occupation of the enzyme active site. IAM inhibits the active site SH− group of FXIIIa and destroys its activity irreversibly. When the plasma was pretreated with EACA or IAM in the presence of ^125^I-factor H before clotting, no large aggregates were seen in both cases in the SDS-PAGE and autoradiography (**Figure 7A**, lanes 3 and 4). Similar results were obtained for C1q (**Figure 7B**, lanes 3 and 4). This suggested that the covalent binding of factor H or C1q diminished when FXIIIa was inhibited, either by EACA or IAM, confirming that the covalent interaction of ^125^I-factor H and ^125^I-C1q with fibrin clots occurred via FXIIIa.

### Complement classical pathway is activated by fibrin clot formation

**Figure 8:**
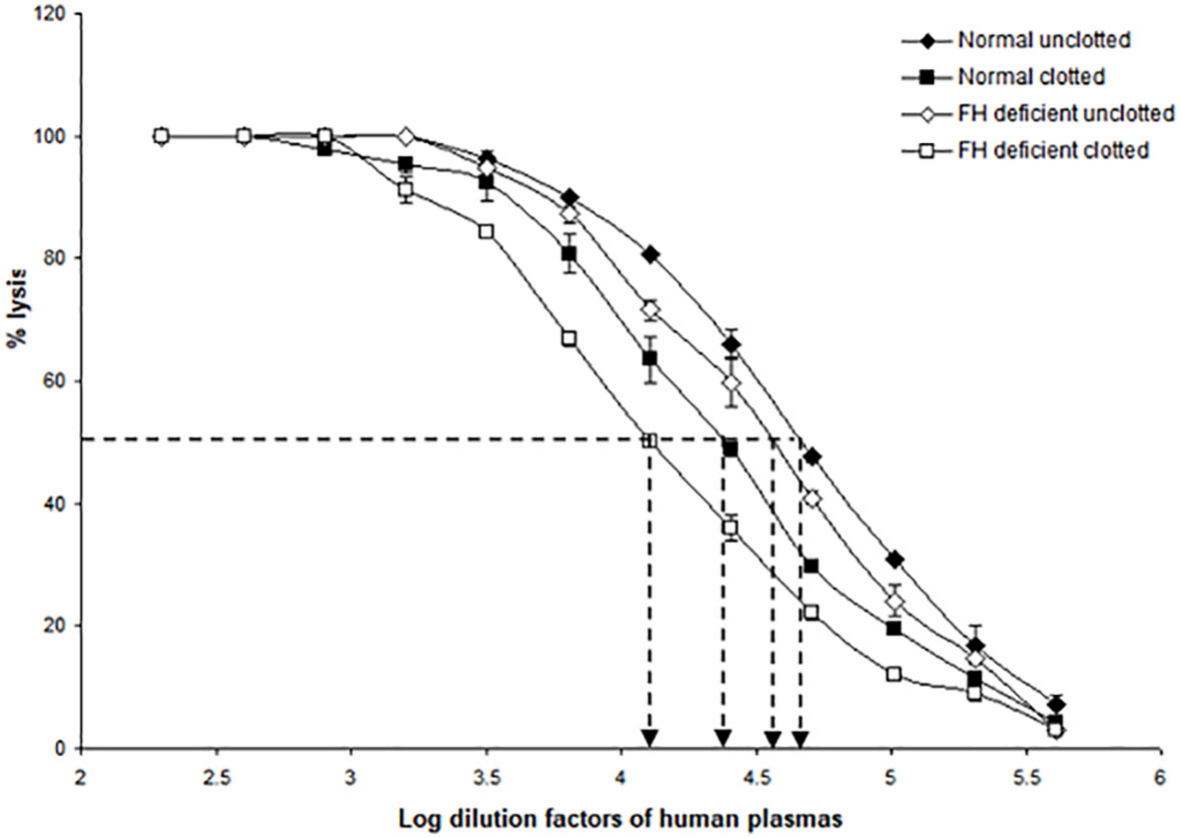
Activation of complement by clot formation in the absence of factor H. Clots were formed in normal plasma and factor H-depleted human plasma, and the supernatants of each sample were tested to assess the remaining complement C4 activity. The supernatants removed from normal and factor H-depleted human plasmas (1/200 up to 1/409,600) were serially diluted in DGVB^2+^, and each dilution was incubated with 1/32 dilution of C4-deficient guinea pig serum and sensitized sheep red blood cells (EA) for 1 h at 37°C. Normal and factor H-depleted plasmas (dilution of 1/200 to 1/409,600) without clotting were also examined for C4 activity. Three independent experiments were performed in duplicate, and the average is shown. C4 consumption by clot-induced complement activation was calculated as follows. The dilutions of the plasmas required to provide 50% lysis were read off from the graph. C4 activity is expressed as the reciprocal dilution of the serum/plasma required to give 50% lysis, which is expressed as units of C4 activity per unit volume. For example, normal human plasma gives 50% lysis at 1:41,687 dilution and can be said to have 41,687 activity units of C4 per unit volume. The amount of C4 consumption by clots was calculated by comparing with that of unclotted plasma (normal human plasma *vs*. normal human plasma after clotting and factor H-depleted plasma *versus* factor H-depleted plasma after clotting). The results demonstrated that, in normal plasma after clotting, there was a decrease in C4 activity by 42.5% compared with normal unclotted plasma. In factor H-depleted plasma, there was a 65.3% decrease in C4 activity after clotting compared with factor H-depleted unclotted plasma. EC_50_ values were calculated (the antilog of the 50% lysis dilution factor) and are listed in **Table 2**.

Since factor H and C1q both bound non-covalently and covalently to the clots formed in plasma, it was of interest to examine whether clots activated the complement system, as would be expected from the binding of C1q, as well as whether factor H regulated the complement activation induced by fibrin clots. To examine the effects of clot formation on complement activation, a hemolytic assay for C4 was carried out with sensitized sheep red blood cells (SRBC) and either normal or factor H-depleted plasmas (in the presence or absence of clotting). This assay, which used C4-deficient guinea pig serum, measures the relative amount of classical pathway activation as determined by C4 consumption. The C4 assay was used because this is specific for the classical and (lectin) pathway and in order to avoid the involvement of C3, as factor H-depleted plasma becomes secondarily depleted of C3. When serially diluted normal and factor H-depleted plasmas were incubated with sensitized EA and C4-deficient guinea pig serum, comparison between the normal and factor H-depleted plasmas showed that there was a small loss of C4 activity in the factor H-depleted plasma through factor H depletion procedures (**Figure 8**). In order to assess the complement activation, possibly induced by clot formation, fibrin clots were initially formed in the presence or absence of factor H using normal or factor H-depleted plasmas. After clotting, the supernatants were assayed for C4 activity (**Table 2**). The results demonstrated that, in normal plasma, there was a decrease in C4 activity by 42.5% after clotting compared with normal unclotted plasma. In factor H-depleted plasma, there was a 65.3% decrease in C4 activity after clotting compared with factor H-depleted unclotted plasma (**Figure 8**). Thus, clots activate the classical pathway of complement in normal and factor H-depleted plasma. Moreover, C4 depletion induced by clotting of the factor H-depleted plasma was approximately 50% greater than that in normal plasma.

**Table 2:** EC_50_ values for the complement consumption assay as depicted in **Figure 8**.

Condition	Log_10_ dilution factor (50% lysis)	EC_50_ (antilog 50% lysis) (U/ml)
Normal unclotted	4.66	45.7 × 10^3^
Normal clotted	4.37	23.4 × 10^3^
FH-deficient unclotted	4.56	36.3 × 10^3^
FH-deficient clotted	4.10	12.6 × 10^3^

FH, factor H.

**Figure 9:**
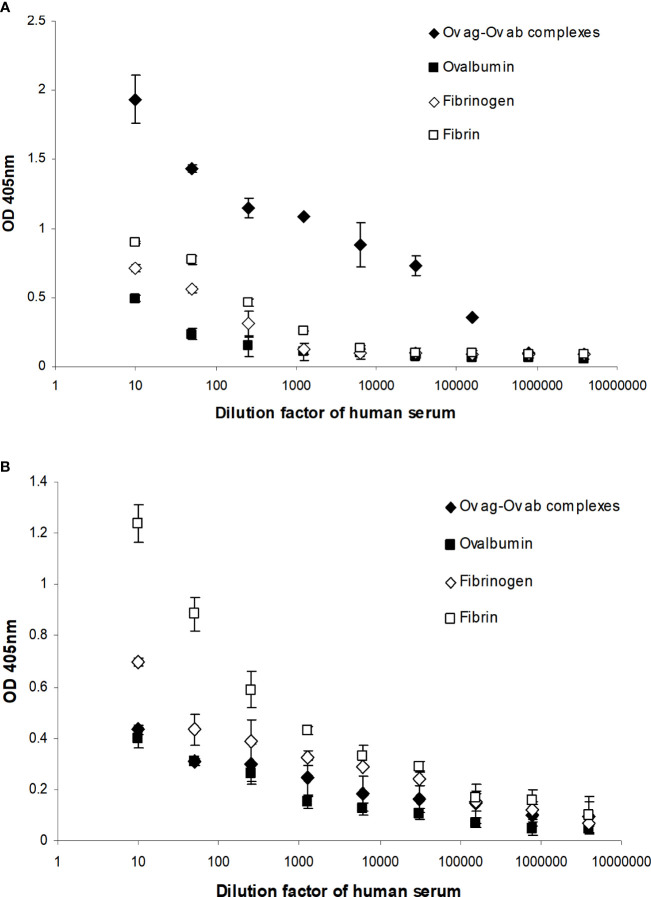
Detection of the membrane attack complex (MAC) on fibrin-coated wells **(A)** and in the supernatants of human serum incubated with the fibrin-coated wells **(B)**. **(A)** Serial dilutions of fresh human serum (1/10, 1/50, 1/250, 1/1,250, 1/6,250, 1/31,250, 1/156,250, 1/781,250, and 1/3,906,250) were incubated with ovalbumin antigen–antibody complex-, ovalbumin-, fibrinogen-, and fibrin-coated wells. After incubation, the supernatant from each well was removed and the well washed three times in PBS–0.1% Tween 20. MAC was detected in ovalbumin antigen–antibody complex-, ovalbumin-, fibrinogen-, and fibrin-coated wells with mouse anti-neo C9 antibody using an ELISA system. **(B)** MAC was also detected in the supernatants in a capture ELISA system with rabbit anti-C9 monoclonal antibody. To measure the nonspecific formation of MAC, serial dilutions of fresh human serum were incubated with blocked wells alone, and the MAC from each well and the supernatant were assayed in the same ELISA and **c**apture ELISA systems as above. These values were subtracted from each sample as a background. The means of three experiments are presented with standard deviations.

Complement activation by fibrin clot formation was also measured by MAC formation as this determines whether the complement classical pathway is fully activated by clots, i.e., up to the C9 stage. This was performed by incubating fibrin-coated wells with serially diluted fresh human serum. The MAC deposited on the fibrin was then measured directly from the fibrin-coated well after removing the human serum. Incubation of the same dilutions of human serum with uncoated wells (blocked with PBS–0.5 mM EDTA and 0.1% Tween 20) was used to measure the nonspecific formation of MAC in the well. This was then subtracted from each sample value. As shown in **Figure 9A**, there was detectable MAC on the fibrin-coated wells when the wells were incubated with 1/10, 1/50, and 1/250 diluted human serum. Fibrinogen-coated wells showed lower MAC deposition with 1/10 dilution, and 1/50 dilution of human serum- and ovalbumin-coated wells also showed a low level of deposition. The OVA ag–ab complex-coated wells were used as a positive control and showed a high level of MAC formation compared with the fibrin- or fibrinogen-coated wells. Thus, fibrin activated complement, which led to the formation of MAC. Interestingly, MAC was detected on the fibrin-coated wells, showing that it could also be bound on the surfaces of fibrin clots. MAC was also directly detected on the OVA ag–ab complex-coated wells. When MAC is formed, it is inserted in the lipid bilayer of the plasma membrane of cellular complement activators. Otherwise, it can react with any of several plasma proteins, e.g., clusterin or S-protein (vitronectin), which prevents insertion into lipid bilayers. Since the wells used in this system had no lipid bilayer, the MAC must be binding to some structure on the fibrin or OVA ag–ab complexes. The MAC–plasma protein complexes (e.g., SC5b-9, the complex with S-protein) might also bind to these surfaces. The assays used did not distinguish between MAC (C5b-9) and the other forms such as SC5b-9. In a capture ELISA system (**Figure 9B**), MAC was measured from each supernatant of serially diluted human serum that was incubated with fibrin-coated wells. There was a high level of MAC in the supernatants from the fibrin-coated wells, a lower level from the fibrinogen-coated wells, and a low level from the OVA ag–ab complex-coated wells. This suggests that both fibrin and immune complexes activate complement to produce MAC. However, for the immune complexes, most of the MAC bound to the complexes, while for fibrin, most of the MAC remained in solution. This confirms that the loss of C4 activity on clotting is due to complement activation and not to the sequestration of inactivated C4 by the clots.

### C1q and factor H bind to the enhanced clots formed in the presence and absence of plasma

**Figure 10:**
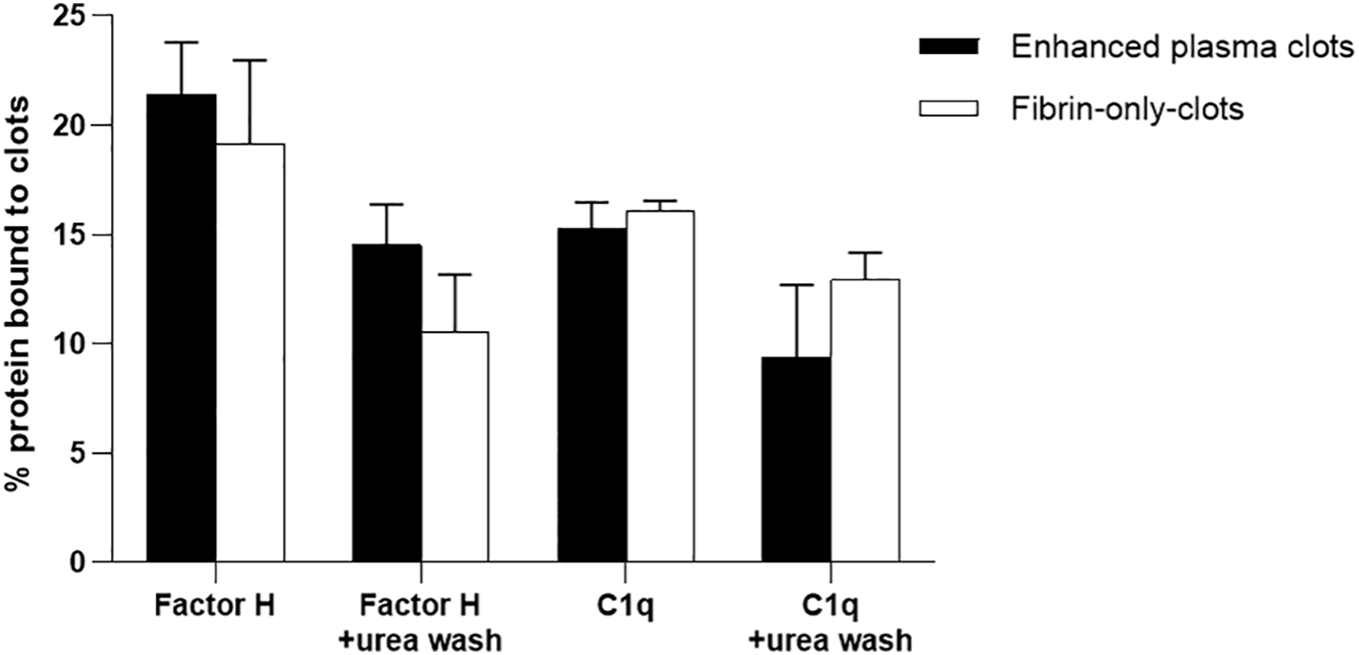
Binding of ^125^I-factor H or ^125^I-C1q to the “enhanced clots” formed in the presence or absence of plasma. Enhanced plasma clots were formed in the presence of ^125^I-factor H or ^125^I-C1q (25,000 cpm/reaction). Clots were washed three times with 500 μl HEPES–1/2-saline buffer or with 10 mM HEPES, 70 mM NaCl, 0.5 mM EDTA, and 5 M urea (pH 7.4). The concentration of fibrinogen in the “enhanced clot” mixture was 3.5 mg/ml. To produce clots in the absence of plasma, 3.5 mg/ml purified fibrinogen in HEPES–1/2-saline buffer was used. The fibrinogen solution (10 μl of 3.5 mg/ml) was premixed with ^125^I-factor H or ^125^I-C1q (25,000 cpm/reaction) in a total volume of 96.5 μl in 10 mM HEPES, 70 mM NaCl and 5 mM CaCl_2_ (pH 7.4). Clotting was initiated by adding 3.5 μl of thrombin (final concentration, 8.75 μg/ml). This quantity of thrombin was selected as the incubation of fibrinogen with thrombin at the same weight ratio resulted in complete cleavage of the α- and β-chains of fibrinogen at 40 min incubation at 37°C, as evidenced by the SDS-PAGE analysis in **Figure 7**. For the enhanced clots, the mixture was incubated for 40 min at 37°C and the resulting clots processed and washed similar to the “enhanced clots.” The ^125^I-factor H or ^125^I-C1q that remained associated with clots was measured. Three independent experiments were conducted in triplicate. *Error bars* represent ±standard deviations. Significance was determined using unpaired *t*-test (non-significance at *p* > 0.05 between the groups of enhanced plasma clots *vs*. fibrin-only clots).

It was further investigated whether the binding of factor H and C1q to fibrin clots is direct or whether it could be mediated via other plasma proteins. The assays used here were fibrin immobilized on microtiter plates and fibrin clots formed in human plasma. However, although we were able to show that factor H bound to fibrin-coated wells, the binding of C1q to fibrin-coated wells was uncertain as it was inhibited by BSA. Moreover, the binding of factor H and C1q to the clots formed in plasma was high, but it was not certain whether factor H and C1q bind directly to fibrin or to the other proteins present in the plasma clots. Therefore, it was necessary to examine the binding to fibrin-only clots. Thus, to consolidate this work, the fibrin-only clots and, as a control, the enhanced plasma clots were used. The results showed that the binding rates of ^125^I-factor H to the enhanced plasma and fibrin-only clots, both at equal concentrations, were 21.5% and 19%, respectively, when the clots were washed with HEPES–1/2-saline (**Figure 10**). The binding of ^125^I-C1q to the enhanced plasma and fibrin-only clots was also observed, and the amounts of ^125^I-C1q bound to both clots were very similar. When the clots were washed with urea buffer, the binding of both ^125^I-factor H and ^125^I-C1q to the enhanced plasma and fibrin-only clots was reduced compared with the clots washed with HEPES–1/2-saline, but for both types of clots, there was still substantial binding of both factor H and C1q. The binding of factor H and C1q to the fibrin in the clots is therefore mainly direct and does not require the presence of other plasma proteins.

### Reduction of factor H and C1q levels in human serum compared with those in plasma

**Figure 11:**
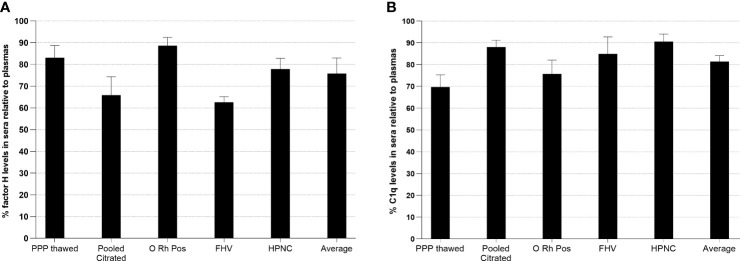
Measurement of relative factor H **(A)** or C1q **(B)** levels in human sera compared with those in plasmas. In order to investigate whether factor H or C1q is bound to clots, the levels of factor H and C1q in plasma and the serum from the same plasma were assayed. Five samples of citrated plasmas with different ages in storage and conditions were tested. Serial twofold dilutions of each plasma and the serum (1/20 to 1/10240) were incubated with monoclonal anti-human factor H antibody-coated wells for 1 h at 20°C. After incubation, the plates were washed with PBS–0.1% Tween 20 and factor H was detected with rabbit–human factor H using an ELISA system. C1q was also measured from the same dilutions of the plasma and the serum used in the ELISA for factor H. Each dilution was incubated with ovalbumin antigen–antibody complex-coated wells for 1 h at 20°C. C1q was detected with biotinylated rabbit anti-human C1q antibody. For each sample, a graph of dilution factors of the plasma and the serum against OD_405 nm_ was drawn. The dilution factors of the plasma and the serum at OD_405nm_ of 2.5 were read off from the graph. The percentage of the level of factor H in the serum was calculated by comparing with that in the plasma (assuming that the level of factor H in the plasma was 100%). The percentage of the level of C1q in each serum sample was also calculated as above. *Average* represents the mean of the relative factor H and C1q levels of five serum samples. *PPP Thawed*, platelet-poor plasma; *O Rh Pos*, O Rhesus-positive plasma; *FHV*, fresh plasma from a healthy volunteer; *HPNC*, human normal plasma citrated. Three independent experiments were conducted in triplicate. Data are represented as a percentage of the level of factor H or C1q in sera relative to plasmas.

In order to examine whether factor H or C1q is bound to clots, the levels of factor H or C1q in plasma and the serum from the same plasma were assayed in an ELISA system. Five samples of citrated plasmas with different ages in storage and conditions were examined (**Figure 11**). The results showed that, in all five samples, the levels of both factor H and C1q were reduced in sera compared with those in the plasmas. In a healthy volunteer sample, approximately 38% of the level of factor H was reduced relative to the plasma and 15% of that of C1q was lost in serum compared with the plasma. Thus, our results support the previous finding that factor H and C1q bind to fibrin clots.

## Discussion

The complement and coagulation systems are evolutionarily and functionally related systems that play crucial roles in maintaining physiological homeostasis and fulfilling distinct yet complementary roles: the complement system is pivotal in clearing cellular debris and defending against pathogens through immune surveillance, while the coagulation pathway is vital for preserving fluid balance and facilitating tissue repair in the aftermath of vascular injuries (43). Furthermore, the complement system also acts as a link between the immune system and the coagulation pathway (64). Multiple studies have revealed that these systems do not function in isolation but are engaged in a dynamic cross-talk, particularly evident in the pathophysiological conditions of various diseases, where they can lead to severe complications such as thrombosis in systemic lupus erythematosus (SLE) and antiphospholipid syndrome (APS) (65), disseminated intravascular coagulation (DIC) (66), or multiple organ failure (MOF) (67) and asthma (68), where their simultaneous activation has been evidenced. Importantly, deficiencies in the negative regulators of the complement system, as observed in conditions such as paroxysmal nocturnal hemoglobinuria (PNH) and atypical familial hemolytic uremic syndrome (aHUS), are linked to an increased risk of thrombosis, highlighting the importance of a finely tuned balance between these pathways (69). The complement system influences thrombus formation mainly by upregulating the inflammatory response and by increasing the clotting efficacy of blood (70). The complement system is known to activate platelets, enhance TF expression across various cell types, modulate mast cell and basophil activity, and, via its MAC, alter the cell membrane phospholipids, triggering the initiation of the TF-mediated coagulation pathway (51, 70–73).

C1q and factor H, which are key regulators in the complement system, exert significant influence over the coagulation cascade. The interaction of C1q with platelet surface receptors not only enhances platelet procoagulant activity through the expression of integrins and P-selectin but also promotes platelet adhesion to activated endothelial cells (74–76). The role of C1q in modulating the expression of TF in adventitial fibroblasts and coronary artery vascular smooth muscle cells via its binding to gC1qR further implicates it in thrombus formation and hemostasis regulation (77). Experimental findings, such as the prolonged bleeding and increased blood loss in C1q-deficient mice compared with wild-type animals, underline the direct involvement of C1q in coagulation processes, although the underlying mechanisms warrant further investigation (78, 79).

Factor H is known to compete with coagulation factors for binding sites on cell surfaces and within the extracellular matrix, potentially altering the local coagulation environment (42, 80). This competition could inhibit the availability of negatively charged phospholipid surfaces that are essential for the assembly of coagulation factor complexes, thus displaying anticoagulant properties (14, 42, 81). Mutations in the factor H gene can lead to uncontrolled complement activation, contributing to the pathogenesis of conditions such as aHUS (82–85), which is characterized by hemolytic anemia, thrombocytopenia, and acute kidney failure, predominantly due to endothelial damage and thrombosis in small vessels. Moreover, polymorphisms in the factor H gene are associated with age-related macular degeneration (AMD), a leading cause of blindness among the elderly (86, 87). Considering the profound and complex interplay between these pathways and the roles of C1q and factor H in influencing the coagulation process, this investigation aimed to further elucidate the impact of clot formation on complement activity. Hence, we investigated whether fibrin, which is the main protein constituent of blood clots, activates complement. Several studies have indicated that both the globular domains and the collagen-like domains of C1q are able to bind fibrinogen, that its receptor (C1q-R) inhibits the polymerization of fibrin (88, 89), and that factor H binds to fibrinogen with high affinity (90). Therefore, we examined whether factor H and C1q interact with fibrin clots using first a microtiter plate assay and later in a more physiological clotting assay. The results obtained in the plate assays demonstrated that factor H and C1q interact with fibrin immobilized on plates, and for both, the binding to fibrin was greater than that to fibrinogen (**Figure 2**). The binding of factor H was shown to be dose-dependent and saturable (**Figure 3**). The Scatchard plots further showed that the affinity of the binding of factor H to fibrin was exceptionally high (*K*_d_ = 55 pM), and there was one major class of binding sites on the fibrin-coated wells. Ionic interactions are important for factor H–fibrin binding, as there was a decrease in interaction with an increase in salt strength. The quantity of factor H and C1q bound to the fibrin plates was, however, very small. This could be due to the limited number of binding sites present in the fibrin-coated wells. It was difficult to envision how fibrin could be made to bind to the plates in a correct physiological configuration (i.e., polymerized); therefore, the presentation of fibrin on the plates used was likely to be very artificial. However, the apparent specificity of the binding of factor H and its preference for fibrin over fibrinogen justified continuing the investigation using a system that more closely resembles physiological conditions. Therefore, clotting of the whole plasma or of the plasma supplemented with extra fibrinogen was used. The results indicated that factor H and C1q do bind to clots and that a large proportion of the bound factor H and C1q might be covalently bound (**Figures 5**, **6**). In contrast to the fibrin-coated wells, the maximal binding of factor H and C1q to fibrin clots was very high (i.e., a high percentage of the total factor H and C1q present in plasma). There was evidence of cross-linking taking place between the fibrin monomers for a period of time as the clot sizes decreased after 96 min incubation, and a further reduction in size (approximately 10-fold) was observed after 16 h incubation. This is likely an effect of the interaction with FXIIIa, which is a critical component in the coagulation pathway that cross-links adjacent COOH and NH_2_ groups in neighboring fibrin molecules (91, 92). The binding of factor H to clots increased up to 16 h incubation, at which time most of the bound factor H molecules were no longer dissociated by urea, i.e., were covalently linked to clots. The binding of C1q was approximately 24% (after 16 h incubation) when the clots were washed with a non-denaturing buffer, but only 18% of the bound C1q remained associated with clots after washing with a denaturant for the same incubation time. Although the binding of C1q was partially dissociable with the denaturant at 16 h incubation, the result indicated that the binding of both factor H and C1q is covalent. SDS-PAGE analysis (**Figure 7**) showed that ^125^I-factor H and ^125^I-C1q were incorporated into a large aggregate, which is likely to be factor H and C1q cross-linked to clots. This evidence was supported by control experiments where no high-molecular-weight aggregates were seen when FXIIIa was inhibited by either EACA or IAM. Therefore, we can conclude that FXIIIa cross-links factor H and C1q into clots. Similar high-molecular-weight complexes were found in a patient with advanced ovarian cancer, indicating its potential clinical significance (93).

FXIII consists of two catalytic A-subunits (FXIIIA) and two non-catalytic B-subunits (FXIIIB) held together by non-covalent bonds. Human FXIIIB is composed of 10 CCP domains, which are the characteristic domains of the regulatory proteins of the complement activation system including factor H. Moreover, a significant structural similarity between FXIIIB and factor H was shown, with a high degree of amino acid identity between the FXIIIB CCP5 and the factor H CCP16 and CCP18 (94). Thus, it is likely that factor H present in the platelets interacts with FXIIIa, which then cross-links into the clots. The proportion of factor H that was covalently bound was very high (approximately 32%) in the clot–urea wash assay (**Figure 6**). In the SDS-PAGE analysis, however, only 5% of the radioactivity was in the large aggregate (**Figure 7**). There are several possibilities for the quantitative differences in the cross-linked factor H analyzed in the clot–urea wash assay and the SDS-PAGE. Firstly, in the clot–urea wash assay, the amount and the concentration of plasma used were larger than those in the SDS-PAGE analysis [i.e., 45% plasma (final concentration in a final volume of 100 μl) was used in the clot–urea wash assay, whereas only 0.9% plasma (final concentration in a final volume of 50 μl) was used in the SDS-PAGE]. Secondly, the amount of extra fibrinogen used in the clot–urea wash assay was higher (200 μg in 100 μl) than that in the SDS-PAGE (20 μg in 50 μl). Thirdly, the SDS-PAGE analysis may have underestimated the amount of factor H covalently bound. Because the factor H cross-linked to clots remained in the loading well, there may have been considerable loss of cross-linked factor H while processing the gel after electrophoresis. However, the results obtained from both assays suggest that factor H binds to clots covalently, and the percentage of factor H covalently linked to clots was high: 5% (obtained from SDS-PAGE) to 32% (obtained from the clot–urea wash assay). The phenomenon of covalent binding may account for the unusually high affinity of factor H for fibrin on the coated plates (**Figure 6**). The very low *K*_d_ could be an artifact generated by some proportion of factor H becoming covalently bound since FXIIIa will also be on the plates just as it is on the clots.

In order to establish that the binding of factor H and C1q is not a simple entanglement, several plasma proteins were assessed for binding to fibrin clots (**Table 1**). Only ^125^I-plasminogen was shown to be bound to clots covalently. This was expected because fibrin is the major plasmin substrate. Plasminogen can bind to fibrin through lysine residues, and it can be converted to plasmin by the tissue plasminogen activator and by urokinase (22). However, the other plasma proteins (e.g., HSA, transferrin, α2-macroglobulin, and IgG) did not interact with clots. These proteins span the size range of C1q and factor H. This suggests that the binding of factor H, C1q, and plasminogen is specific and is not a simple physical entanglement. The SDS-PAGE analysis (**Figure 7**) further showed that FXIIIa is essential for the covalent binding of factor H and C1q to clots.

The binding of both factor H and C1q to the fibrin-coated plates was low, but that to clots was very high, which can be explained by several factors: i. The fibrin clots made on plates were “artificial” and randomly oriented. The fibrin clots were produced on the plates by adding fibrinogen and, subsequently, thrombin, which can limit the mobility of fibrinogen that is necessary for its cleavage by thrombin. This can produce an unknown extent of conversion of fibrinogen to fibrin. ii. In clots, other proteins that mediate binding may be present, and thus it is possible that the binding may not be to fibrin. As shown in **Figure 10**, however, the binding of factor H and C1q to fibrin in clots was mainly direct. iii. The surface area of the clots is likely to be very large compared with the plate, but this is difficult to measure. The quantity of fibrin in clots was much greater than that on plates. iv. In order to optimize clot formation and the potential binding of ^125^I-factor H or ^125^I-C1q to fibrin clots, fibrinogen (2 mg/ml) was added to the reaction mixture prior to clot formation (referred to as “enhanced clots”). Thus, this increased the surface area of clots for factor H or C1q binding, which in turn increased the concentration of “epitopes” for factor H binding. v. The covalent linking of factor H and C1q in clots by FXIIIa (the concentration of FXIII in plasma was 10 μg/ml) made binding irreversible so that no dissociation can occur; therefore, the apparent avidity of factor H or C1q was increased.

Previous studies have established that factor H can regulate the classical pathway activation triggered by certain targets (12–14). Notably, factor H has been found to compete with C1q for binding to cardiolipin, a known activator of the classical pathway, thereby preventing its activation (14). Thus, factor H can act as a control switch for C1q-mediated complement activation, thereby preserving immune homeostasis. Similarly, the binding of C1q to lipid A, a component of the outer membrane of Gram-negative bacteria, typically initiates activation of the classical pathway (13). However, factor H can outcompete C1q in this context as well, effectively regulating the immune response toward non-self-entities such as bacteria. Moreover, factor H also has implications in the clearance of apoptotic cells, or dying cells, by phagocytes, a process that is typically enhanced by C1q (12, 95). It appears that factor H can modulate the C1q-enhanced uptake of apoptotic cells, possibly by acting as a bridge between apoptotic cells and phagocytes. This role of factor H could potentially modify signaling pathways to reduce the pro-inflammatory effects often associated with the process, adding yet another layer of immune regulation. By outcompeting C1q in binding to both self and non-self-ligands and in controlling the phagocytosis of apoptotic cells, factor H helps maintain a balanced immune response and protects against potential self-damage.

Here, we investigated the complement classical pathway activation by clots and a possible role for factor H in its downregulation. In order to measure the classical pathway activation, a C4 consumption assay was performed using normal human plasma or factor H-depleted plasma. The results indicated that clots activated complement both in normal and factor H-depleted plasma (**Figure 8**). In addition, the assays for MAC deposition also demonstrated that complement was activated by clots fully up to the C9 stage and that the reaction was completed by the formation of MAC (**Figure 9**). Thus, the C4 consumption by clots was due to C4 activation, not just C4 binding to the clots. Moreover, there was an increase in C4 consumption by clots in the factor H-depleted plasma compared with the normal plasma. This suggests that, in the absence of factor H, C4 consumption by the classical pathway triggered by clots is higher by 50% than in normal plasma. These results support the hypothesis that factor H downregulates the activation of the classical pathway, possibly by competing with C1q for binding to fibrin clots. However, we were unable to demonstrate a direct competition between C1q and factor H in this clotting assay because the concentration of factor H present in the plasma was high (between 150 and 750 μg/ml) so that it was not practical to outcompete the binding of factor H to fibrin clots by C1q. Moreover, we could not perform an assay to measure the direct competition between C1q and factor H for fibrin using a plate binding assay because the specificity of the binding of C1q to the fibrin-coated wells was not certain as the binding was inhibited by BSA. Furthermore, the binding of factor H to fibrin coated on microtiter plates was only detectable at a low salt strength (20 mM NaCl), and at this salt strength, we found that factor H and C1q interact with each other (data not shown). However, the experimental conditions used did not account for the effect of the lectin pathway; as such, the pattern recognition molecules of the lectin pathway may have also contributed to the effect observed.

The results in this study further our understanding of diseases, where the complement and the coagulation systems play a crucial role in the disease pathology, such as COVID-19 and familial HUS. Familial HUS has been extensively considered in terms of alternative pathway activation, which might be increased due to factor H abnormalities or partial deficiency, which leads to hypocomplementemia (i.e., consumption of C3 and factor B) (96). In addition to the general role of factor H in the alternative pathway, here, we wish to emphasize that factor H plays a downregulatory role in the classical pathway at the site of a fibrin clot. This could provide an important linkage to some clinical observations in patients with familial HUS and factor H deficiency. Patients with familial HUS could intermittently exhibit symptoms of intravascular coagulation, hemolytic anemia, thrombocytopenia, and acute renal failure (97). Current studies regarding the pathogenesis of HUS have focused on hemolytic anemia and intravascular coagulation. This study suggests that the binding of factor H is essential and that this interaction could regulate complement activation on the clots. Diminished factor H binding to clots due to functional factor H deficiency in HUS could promote uninhibited complement activation. Factor H can also be considered as an essential regulatory protein in other diseases, e.g., membranoproliferative glomerulonephritis III, where the glomerular deposition of immune complexes was found to be associated with factor H deficiency. Severe acute respiratory syndrome coronavirus 2 (SARS-CoV-2) infection, which was responsible for the COVID-19 pandemic, has been associated with a multisystem and multi-organ inflammatory response that contributes to the high mortality rates in affected individuals (98). Central to this response is the hyperactivation of both the complement and coagulation systems (99–101). Serum samples from patients with COVID-19 revealed a consistent and sustained activation of the complement system. It has been found to play a significant role in the hyper-inflammation and thrombotic microangiopathy observed in severe COVID-19 cases (98). SARS-CoV-2 has the ability to trigger the activation of all three pathways of the complement system: classical, alternative, and lectin pathways (102–104). Furthermore, SARS-CoV-2 was also found to promote the synthesis and release of complement factors from infected respiratory epithelial and endothelial cells through Janus kinase 1 (JAK1)-dependent and/or JAK2-dependent pathways, in addition to the release of procoagulant (clot-promoting) factors (105, 106). The activation of the complement system, specifically the cleavage of C5, can result in the release of prothrombotic factors (106). In addition, C5 cleavage can increase the expression of P-selectin on endothelial cells and trigger TF activation, both of which facilitate the recruitment of inflammatory leukocytes (106). TF can be expressed on neutrophil extracellular traps (NETs), which are released by neutrophils during an immune response (99). This expression of TF can contribute to clotting, a condition that is often associated with severe cases of COVID-19 (107, 108). This is one of the mechanisms by which a hyperactivated complement system could initiate coagulation or blood clotting. Furthermore, TF activation has been reported in the presence of SARS-CoV-2, and there is evidence that TF expression can be disrupted by inhibiting the complement component C3, suggesting that the complement axis plays a critical role in COVID-19-induced inflammation (109). The coagulation system also plays a pivotal role in the pathology of COVID-19. The interaction of the spike glycoproteins of SARS-CoV-2 with the ACE2 receptors on the host cell surface induces to injury in the vascular wall of blood vessels, leading to coagulation and clotting cascade activation and subsequent formation of internal blood clots (99). Furthermore, severe COVID-19 is associated with coagulopathies exacerbated by the formation of NETs, which can block pulmonary lymphatic vessels (110). These clots are often associated with systemic inflammation, disease severity, comorbidities, and increased mortality risk (111). Thus, the activation of the complement system by SARS-CoV-2, along with the formation of clots, presents a significant risk factor in severe COVID-19 cases.

Hence, the reported interactions between the complement and coagulation systems and their dysfunction could play a crucial role in several pathologies. However, further research is needed to fully elucidate these mechanisms and their implications for treatment options.

## Conclusions

This study provides significant insights into the complex interplay between the coagulation and complement pathways, specifically the interaction between factor H, C1q, and fibrin clots (**Figure 1**). Our results indicate that factor H and C1q specifically bind to fibrin clots, with a considerable proportion being covalently bound. Moreover, it was found that clots activated the classical pathway and that factor H can restrict this activation, supporting the idea of factor H having a regulatory role in the classical pathway. The work also suggests that FXIIIa, a critical component in the coagulation pathway, cross-links factor H and C1q into clots. These findings offer a new understanding of how these molecules interact during coagulation and complement activation, which could be pivotal in pathophysiological conditions where these processes are dysregulated, such as COVID-19, hence raising the possibility of therapeutic interventions targeting these molecular relationships.

## Data availability statement

The original contributions presented in the study are included in the article/supplementary material. Further inquiries can be directed to the corresponding author.

## Author contributions

Y-HK: Data curation, Formal analysis, Investigation, Methodology, Validation, Writing – original draft. PV: Data curation, Formal analysis, Software, Visualization, Writing – review & editing. AA: Writing – original draft, Formal analysis, Software. KP: Data curation, Formal analysis, Software, Visualization, Writing – review & editing. UK: Conceptualization, Funding acquisition, Investigation, Methodology, Project administration, Writing – original draft, Writing – review & editing. RS: Conceptualization, Funding acquisition, Investigation, Methodology, Project administration, Writing – original draft.
